# Efficacy of Virtual Care for Depressive Disorders: Systematic Review and Meta-analysis

**DOI:** 10.2196/38955

**Published:** 2023-01-09

**Authors:** Crystal Edler Schiller, Julianna Prim, Anna E Bauer, Linda Lux, Laura Claire Lundegard, Michelle Kang, Samantha Hellberg, Katherine Thompson, Theresa Webber, Adonay Teklezghi, Noah Pettee, Katherine Gaffney, Gabrielle Hodgins, Fariha Rahman, J Nikki Steinsiek, Anita Modi, Bradley N Gaynes

**Affiliations:** 1 Department of Psychiatry University of North Carolina School of Medicine University of North Carolina at Chapel Hill Chapel Hill, NC United States

**Keywords:** depression, virtual, treatment, therapy, efficacy, virtual care, meta-analysis, review, mental health, depressive disorder, virtual intervention, digital intervention, digital health, eHealth, health outcome, digital mental health, health intervention

## Abstract

**Background:**

The COVID-19 pandemic has created an epidemic of distress-related mental disorders such as depression, while simultaneously necessitating a shift to virtual domains of mental health care; yet, the evidence to support the use of virtual interventions is unclear.

**Objective:**

The purpose of this study was to evaluate the efficacy of virtual interventions for depressive disorders by addressing three key questions: (1) Does virtual intervention provide better outcomes than no treatment or other control conditions (ie, waitlist, treatment as usual [TAU], or attention control)? (2) Does in-person intervention provide better outcomes than virtual intervention? (3) Does one type of virtual intervention provide better outcomes than another?

**Methods:**

We searched the PubMed, EMBASE, and PsycINFO databases for trials published from January 1, 2010, to October 30, 2021. We included randomized controlled trials of adults with depressive disorders that tested a virtual intervention and used a validated depression measure. Primary outcomes were defined as remission (ie, no longer meeting the clinical cutoff for depression), response (ie, a clinically significant reduction in depressive symptoms), and depression severity at posttreatment. Two researchers independently selected studies and extracted data using PRISMA (Preferred Reporting Items for Systematic Reviews and Meta-Analyses) guidelines. Risk of bias was evaluated based on Agency for Healthcare and Research Quality guidelines. We calculated odds ratios (ORs) for binary outcomes and standardized mean differences (SMDs) for continuous outcomes.

**Results:**

We identified 3797 references, 24 of which were eligible. Compared with waitlist, virtual intervention had higher odds of remission (OR 10.30, 95% CI 5.70-18.60; N=619 patients) and lower posttreatment symptom severity (SMD 0.81, 95% CI 0.52-1.10; N=1071). Compared with TAU and virtual attention control conditions, virtual intervention had higher odds of remission (OR 2.27, 95% CI 1.10-3.35; N=512) and lower posttreatment symptom severity (SMD 0.25, 95% CI 0.09-0.42; N=573). In-person intervention outcomes were not significantly different from virtual intervention outcomes (eg, remission OR 0.84, CI 0.51-1.37; N=789). No eligible studies directly compared one active virtual intervention to another.

**Conclusions:**

Virtual interventions were efficacious compared with control conditions, including waitlist control, TAU, and attention control. Although the number of studies was relatively small, the strength of evidence was moderate that in-person interventions did not yield significantly better outcomes than virtual interventions for depressive disorders.

## Introduction

Prior to the COVID-19 pandemic, the lifetime prevalence of major depressive disorder (MDD) was over 20% for adults in the United States [1], and the majority (71%) of cases were untreated [2]. Compared with the prepandemic period, depressive symptoms became over three times more prevalent [3] during the pandemic, with up to 48% of citizens of developed nations reporting clinically significant depression [4]. At the same time, pandemic constraints critically challenged the provision of mental health services. Cost-effective, scalable, affordable, and accessible interventions were urgently needed, and the use of virtual care expanded quickly [5]. However, the efficacy of modern virtual interventions had not been systematically examined. Thus, the aim of this systematic review and meta-analysis was to fill this gap to inform clinical, administrative, and policy decision-making.

Prior systematic reviews and meta-analyses examined the evidence supporting the efficacy of computerized or virtual cognitive behavioral therapy (CBT) for MDD or depressive symptoms compared with no treatment or treatment as usual (TAU) (ie, referring participants to primary care providers or other health clinics in their local community to manage their depressive symptoms). Moreover, meta-analyses [6] and umbrella summaries across meta-analyses [7] have suggested that virtual treatment works at least as well as in-person treatment for those with depressive symptoms. Prior meta-analyses of virtual treatments for adults included studies conducted before 2016, and many included adults with depressive symptoms or various depression and anxiety diagnoses [6,8-10]. Since 2016, individual studies of virtual interventions have proliferated, expanding beyond CBT [11,12], and increased in rigor. As a result, a comparison of modern virtual interventions with not only waitlist or TAU but also with face-to-face interventions [6,13] for adults with MDD is feasible and warranted given that face-to-face psychotherapy had become impractical and, in certain settings, impossible.

Information evaluating whether virtual care is an efficacious alternative to individual, face-to-face intervention with a therapist is needed for clinicians, health systems, payers, and policymakers. In addition, data to guide decisions about which existing virtual interventions are most efficacious for treating depressive disorder are lacking. In the absence of such data, common assumptions about the superiority of in-person treatment have guided clinical decisions and policies regarding depression treatment.

The purpose of this systematic review was to answer three clinically relevant key questions (KQs) for depressive disorders (ie, MDD, persistent depressive disorder, or dysthymia) based on studies conducted in the last 10 years.

KQ1: Does virtual intervention provide better clinical outcomes than no treatment, TAU, or attention control, defined as a rigorous control condition that simulates active treatment without the active ingredient (ie, does it work)?

KQ2: Does in-person intervention provide better outcomes than virtual intervention (ie, is in-person intervention better)?

KQ3: Does one type of virtual intervention provide better outcomes than another type of virtual intervention (ie, what works best)?

The KQs were structured based on Agency for Healthcare and Research Quality (AHRQ) guidance for decision-making related to best practices in treatment [14].

## Methods

### Design

We used the Cochrane Handbook for Systematic Reviews of Interventions methods [15] and AHRQ guidance for grading the strength of evidence [16]. The protocol for this systematic review and meta-analysis is published in the Open Science Framework [17]. Reporting conforms to the PRISMA (Preferred Reporting Items for Systematic Reviews and Meta-Analyses) guidelines [18].

### Search Strategy and Selection Criteria

For this systematic review and meta-analysis, we searched the PubMed, EMBASE, and PsycINFO databases for trials published from January 1, 2010, to October 30, 2021, for MeSH (Medical Subject Headings) and major headings listed in Table A1 of Multimedia Appendix 1. Relevant systematic reviews and meta-analyses were used to identify additional existing literature, and ClinicalTrials.gov was searched to identify unpublished trials.

The study criteria were selected to inform clinical decision-making and policy in the United States. Eligible studies were randomized controlled trials (RCTs) of adults with a clinical diagnosis of MDD, dysthymic disorder, or persistent depressive disorder that tested a virtual psychological intervention for depression in at least one study arm, reported an outcome using a validated depression measure (see Table A2 of Multimedia Appendix 1), and were conducted in countries with a very high human development index (see Table 3 of Multimedia Appendix 1 for a list of eligible countries, [19]). To ensure generalizability of the results to individuals with major depression with access to current technology in the United States, we included studies conducted in similarly highly developed nations. To ensure comparability across studies, we included studies with standard, validated measures of depression, both self-reported and clinician-rated. Because evidence-based treatments for depression differ for children and adults, we excluded studies of children from this review.

References identified through searches were imported into Covidence Systematic Review software (Veritas Health Innovation, Melbourne, Australia). Two reviewers independently screened the titles and abstracts of all references according to the inclusion and exclusion criteria. Studies included by either reviewer were retrieved for full-text screening by two independent reviewers for eligibility. Discrepancies between reviewers were resolved through discussions and consensus.

### Data Extraction

One author extracted summary data from the included trials into standardized forms, and a second senior author (BNG, CES, or LL) checked the data for accuracy. Two authors independently rated the risk of bias across nine domains (see Table A4 in Multimedia Appendix 1) using the Cochrane Risk of Bias tool for RCTs [20] modified for psychotherapy outcome research [21]. Disagreements were resolved by discussion and consensus. Trials with a high risk of bias were excluded, although sensitivity analyses were performed to determine the impact on the results (see Figure A1 in Multimedia Appendix 1).

### Data Synthesis and Analysis

Primary outcomes were rates of remission (ie, no longer meeting the clinical cutoff for depression), rates of response (ie, a clinically significant reduction in depressive symptoms), and depression severity at posttreatment. We calculated odds ratios (ORs) with 95% CIs for remission and response, and calculated standardized mean differences (SMDs, Cohen *d*) with 95% CIs for differences in symptom severity between groups. Forest plots were generated for all outcomes with sufficient data.

To determine the appropriateness of quantitative analyses, the senior authors (BNG, CES, LL, AEB) assessed the clinical and methodological heterogeneity of studies under consideration [15]. We performed meta-analyses using the *meta* package (v 4.19-2) in R version 3.6.1 [22] when two or more trials reported data on outcomes of interest with low levels of heterogeneity. Effect sizes were weighted by their inverse variance. To account for variability in the different study populations, we used random-effects models to estimate pooled or comparative effects with three or more studies. However, because the effect estimates from smaller studies (which are generally more prone to bias) are more influential in random-effects models, we used fixed-effects models in analyses with fewer than three studies to ensure that a small study would not overinfluence the estimates [22].

Statistical heterogeneity in effects between studies included in each meta-analysis was assessed by calculating the *χ*^2^ statistic (*Q*) and the *I*^2^ statistic, assessing the proportion of variation in study estimates due to heterogeneity rather than sampling error [15]. In instances of high heterogeneity, we performed sensitivity analyses to determine the extent to which excluding dissimilar studies changed the overall effect estimates. Most studies had only two study arms (ie, intervention and control); however, two studies had two intervention arms in addition to a waitlist control arm [23,24]. A two-level model was used if there was no significant difference between the three-level model and a two-level model based on a likelihood ratio test [22]. Using AHRQ guidelines [16], we assessed the overall strength of evidence (SOE) considering four factors: directness, consistency, precision, and bias. SOE was assessed by one author (LL) and checked for consensus with two other authors (BNG and CES). We began each SOE assessment with a rating of high and downgraded for factors that reduced the level of confidence. The resulting definitions of high, moderate, low, and insufficient SOE grades are summarized in Table A5 of Multimedia Appendix 1.

## Results

### Characteristics of Included Studies

**Figure 1:**
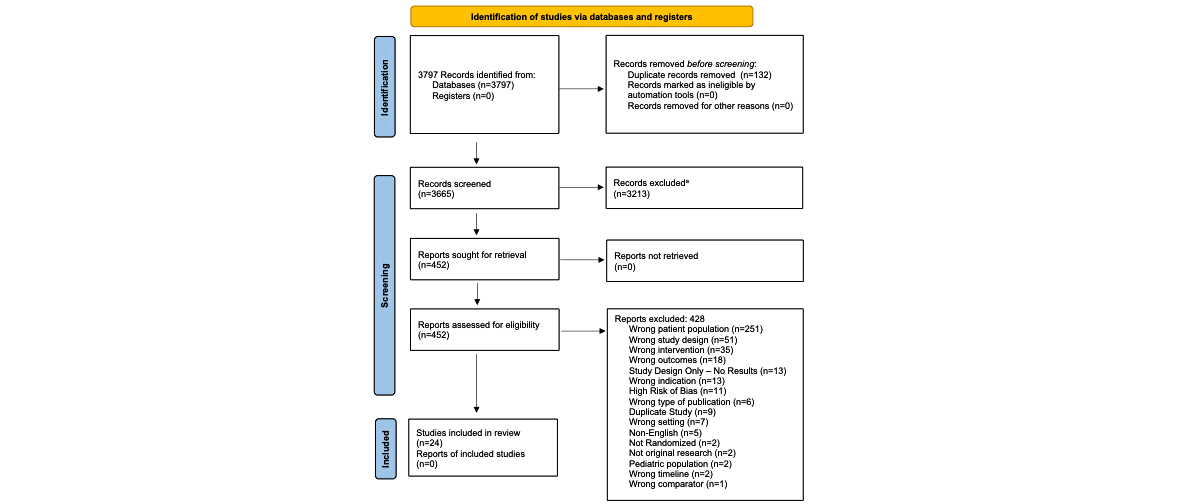
Study selection. *See Table A2 in Multimedia Appendix 1.

Database and manual searching yielded 3797 citations for consideration (Figure 1), 24 of which met the criteria for inclusion in this review. The characteristics of participants included in each study are summarized in Table 1. Participants in all of the included trials were diagnosed with MDD, and those with severe psychiatric comorbidities such as any psychotic disorder or active substance use disorder, bipolar disorder, or acute risk of suicidality were excluded.

**Table 1:** Participant characteristics in each included trial.

Reference^a^		MDD^b^ diagnostic measure^c^	Average MDD severity at baseline	Intervention condition		Comparison condition		Age (years), mean (SD)	Women, n (%)	Some college^d^, n (%)
				Participants, n	Completed posttreatment assessment, n (%)	Participants, n	Completed posttreatment assessment, n (%)			
**KQ^e^1a (virtual vs waitlist)**										
	Berger et al [23]	MINI^f^	Moderate	25	22 (88)	26	22 (85)	39 (14)	36 (70)	32 (63)
	Berger et al [23]	MINI	Moderate	25	25 (100)	26	22 (85)	39 (14)	36 (70)	32 (63)
	Carlbring et al [25]	SCID^g^	Moderate	40	40 (100)	40	38 (95)	44 (14)	66 (83)	61 (76)
	Chan et al [11]	Clinical interview	Moderate	167	109 (65)	153	144 (94)	27 (7)	234 (73)	288 (90)
	Johansson et al [26]	SCID	Moderate	27	25 (91)	27	27 (100)	39 (NR^h^)	31 (57)	23 (42)
	Kenter et al [27]	CIDI^i^	Moderate	136	96 (69)	133	89 (67)	38 (11)	145 (54)	110 (41)
	Smith et al [28]	MINI	Moderate	61	36 (59)	68	55 (81)	40 (13)	106 (82)	89 (69)
	Vernmark et al [23]	SCID	Moderate	30	29 (97)	29	29 (100)	37 (13)	40 (68)	48 (82)
	Vernmark et al [24]	SCID	Moderate	29	27 (93)	29	29 (100)	37 (13)	39 (68)	48 (82)
**KQ1b (virtual vs TAU^j^)**										
	Dennis et al [12]^k^	SCID	Moderate	120	104 (87)	121	100 (83)	31 (6)	241 (100)	181 (75)
	Forsell et al [29]	SCID	Moderate	22	21 (95)	20	18 (90)	30 (5)	42 (100)	30 (71)
	Hallgren et al [30]	MINI	Moderate	317	273 (86)	312	256 (82)	43 (12)	472 (75)	390 (62)
	Löbner et al [31]	ICD-10^l^	Mild to moderate	320	259 (81)	327	307 (94)	44 (13)	446 (69)	NR
	Moreno et al [32]	MINI	Moderate	80	74 (93)	87	85 (98)	44 (12)	149 (89)	28 (17)
	Pfeiffer et al [33]	Medical record	Moderate	167	109 (65)	163	129 (79)	52 (15)	66 (20)	281 (85)
	Raevuori et al [34]	ICD	Moderate	63	57 (90)	61	51 (84)	25 (NR)	90 (73)	NR
	Wozney et al [35]^k^	SCID	Moderate to severe	32	26 (81)	30	24 (80)	29 (5)	62 (100)	14 (22)
**KQ1c (virtual vs attention control)**										
	Flygare et al [36]	SCID	Moderate	48	36 (74)	14	11 (76)	45 (12)	47 (76)	NR
	Johansson et al [37]^m^	MINI	Moderate	46	42 (91)	46	46 (100)	47 (14)	64 (70)	77 (84)
	Ly et al [38]^m^	MINI	Moderate	40	36 (90)	41	36 (88)	36 (11)	57 (70)	51 (63)
	Oehler et al [39]	MINI	Mild to moderate	173	125 (72)	174	127 (73)	42 (12)	274 (79)	229 (66)
	Reins et al [40]	SCID	Moderate	65	49 (75)	66	53 (80)	42 (11)	100 (76)	94 (72)
**KQ2 (in-person vs virtual)**										
	Andersson et al [41]	SCID	Moderate	36	33 (92)	33	32 (97)	42 (14)	54 (78)	NR
	Egede et al [42]	SCID	Moderate	121	106 (88)	120	108 (90)	64 (5)	5 (2)	NR
	Mohr et al [43]	HAMD^n^	Moderate	162	141 (87)	163	151 (93)	NR (NR)	NR	NR
	Thase et al [13]	SCID	Moderate	77	67 (87)	77	66 (86)	46 (14)	102 (66)	152 (99)

^a^Each row represents an intervention arm. Some references are listed more than once because they provided data from multiple intervention arms.

^b^MDD: major depressive disorder.

^c^Participants of all trials were diagnosed with MDD.

^d^Some college means any self-reported level of educational attainment greater than high school or equivalent.

^e^KQ: key question.

^f^MINI: Mini International Neuropsychiatric Interview.

^g^SCID: Structured Clinical Interview for Axis-I Disorders.

^h^NR: not reported.

^i^CIDI: Composite International Diagnostic Interview.

^j^TAU: treatment as usual.

^k^All participants were diagnosed with MDD with perinatal onset.

^l^ICD-10: International Classification of Diseases, 10th revision.

^m^Included in systematic review but excluded from meta-analysis due to differences in methods from other studies.

^n^HAMD: Hamilton Rating Scale for Depression.

The characteristics of each trial, including the length of intervention, treatment modality and mode, provider type, and comparison condition, are summarized in Table 2.

**Figure 2:**
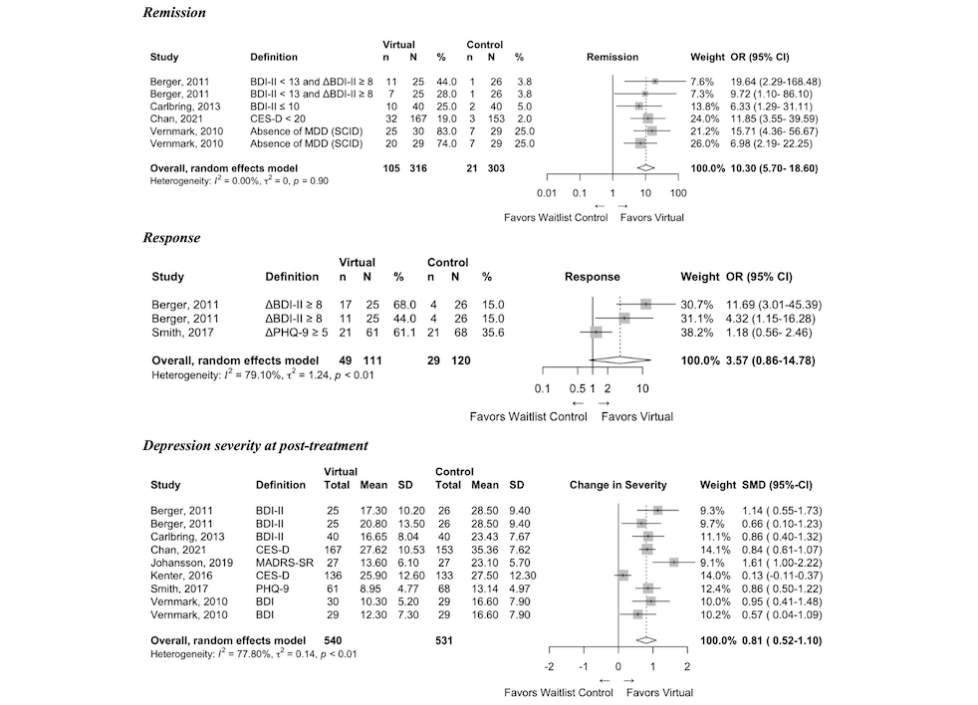
Forest plots of virtual intervention compared with waitlist control clinical outcomes. ΔBDI: Change in Beck Depression Inventory Score; ΔPHQ: Change in Patient Health Questionnaire-9 Score; BDI: Beck Depression Inventory; CES-D: Center for Epidemiologic Studies Depression Scale; MADRS-SR: Montgomery–Åsberg Depression Rating Scale – Self-Report Questionnaire; MDD: Major Depressive Disorder; PHQ-9: Patient Health Questionnaire-9.

**Figure 3:**
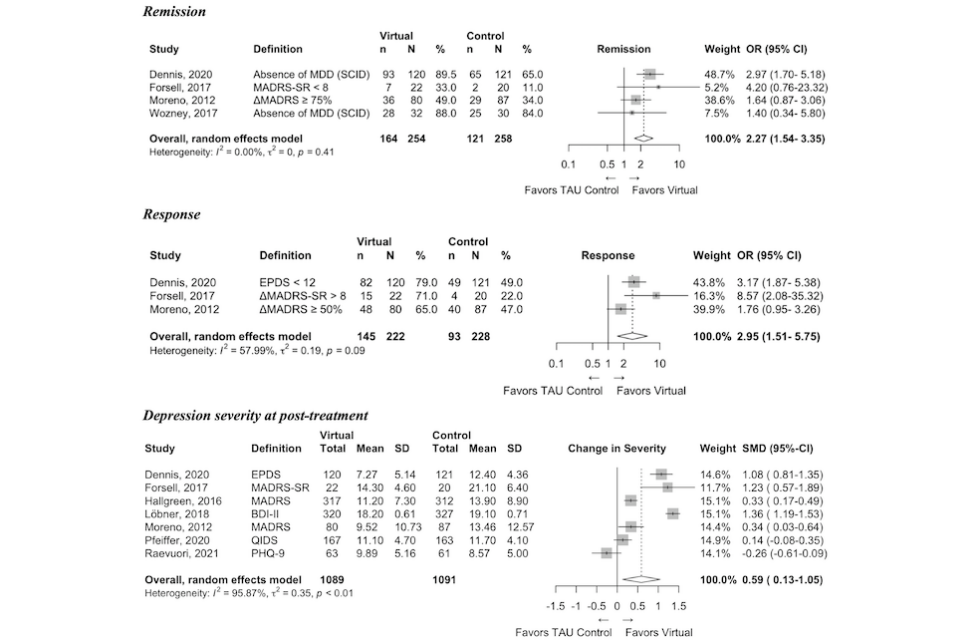
Forest plots of virtual intervention compared with treatment as usual (TAU) clinical outcomes. ΔMADRS: Change in Montgomery–Åsberg Depression Rating Scale Score; ΔMADRS-SR: Change in Montgomery–Åsberg Depression Rating Scale – Self-Report Questionnaire Score; BDI: Beck Depression Inventory; EPDS: Edinburgh Postnatal Depression Scale; MDD: Major Depressive Disorder; MADRS: Montgomery–Åsberg Depression Rating Scale Interview; MADRS-SR: Montgomery–Åsberg Depression Rating Scale – Self-Report Questionnaire; PHQ-9: Patient Health Questionnaire-9.

**Figure 4:**
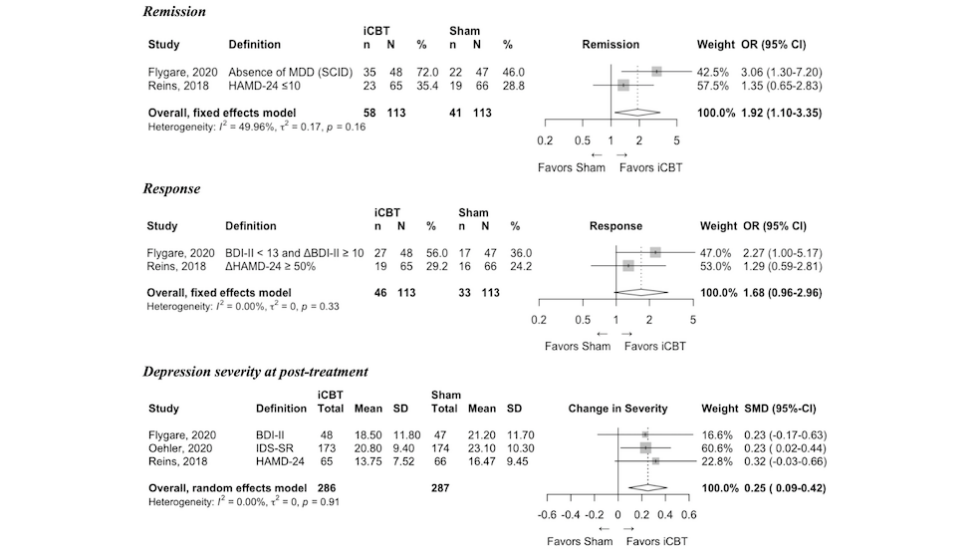
Forest plots for virtual intervention (internet-based cognitive behavioral therapy [iCBT]) compared with virtual sham intervention clinical outcomes. ΔBDI: Change in Beck Depression Inventory Score; BDI: Beck Depression Inventory; HAMD: Hamilton Depression Rating Scale; IDS-SR: Inventory for Depressive Symptomatology – Self-Report; MDD: Major Depressive Disorder; SCID: Semi-Structured Clinical Interview for DSM Disorders.

**Figure 5:**
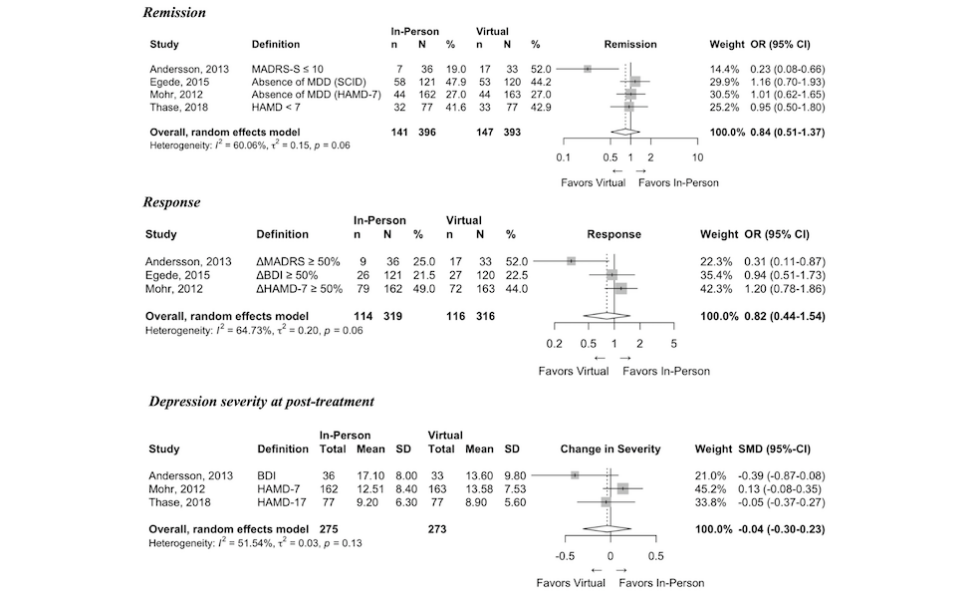
Forest plots for in-person intervention compared with virtual intervention clinical outcomes (key question 2). ΔBDI: Change in Beck Depression Inventory Score; ΔHAMD: Change in Hamilton Depression Rating Scale Score; ΔMADRS: Change in Montgomery–Åsberg Depression Rating Scale Score; BDI: Beck Depression Inventory; HAMD: Hamilton Depression Rating Scale; MDD: Major Depressive Disorder; SCID: Semi-Structured Clinical Interview for DSM Disorders.

Risk of bias assessments across the nine individual domains and an overall summary is presented for each study in Table A4 of Multimedia Appendix 1; detailed information on intervention outcomes is presented in Figures 2-5; and SOE ratings alongside a summary of results are presented in Table 3.

**Table 2:** Trial characteristics.

Reference^a^		Length of intervention (weeks)	Intervention condition			Comparison condition		
			Modality	Mode	Provider type	Modality	Mode	Provider
**KQ^b^1a (virtual vs waitlist)**								
	Berger et al [23]	10	CBT^c^ (Deprexis)	Online intervention, guided	Mental health specialist	Waitlist	NA^d^	None
	Berger et al [23]	10	CBT (Deprexis)	Online intervention, unguided	None	Waitlist	NA	None
	Carlbring et al [25]	7	ACT^e^/BA^f^	Online intervention, guided	Mental health specialist	Waitlist	NA	None
	Chan et al [11]	6	CBT-I	Smartphone intervention, unguided	None	Waitlist	NA	None
	Johansson et al [26]	8	CBT	Online intervention, guided	Mental health specialist	Waitlist	NA	None
	Kenter et al [27]	8	Problem-solving therapy	Online intervention, guided	Student	Waitlist	NA	None
	Smith et al [28]	12	CBT	Online intervention, guided	Mental health specialist	Waitlist	NA	None
	Vernmark et al [24]	8	CBT	Individualized email therapy	Mental health specialist	Waitlist	NA	None
	Vernmark et al [24]	8	CBT	Online intervention, guided	Mental health specialist	Waitlist	NA	None
**KQ1b (virtual vs TAU^g^)**								
	Dennis et al [12]	12	IPT^h^	Telehealth (telephone)	Nurses	TAU	In-person	Nurse
	Forsell et al [29]	10	CBT	Online intervention, guided	Mental health specialist	TAU	In-person	OBGYN^i^
	Hallgren et al [30]	12	CBT	Online intervention, guided	Mental health specialist	TAU	In-person	PCP^j^
	Löbner et al [31]	6	CBT (Moodgym)+TAU	Online intervention, self-guided	PCP	TAU	In-person	PCP
	Moreno et al [32]	24	Supportive therapy+medication	Telehealth (video)	Mental health specialist	TAU	In-person	PCP
	Pfeiffer et al [33]	12	CBT (Beating the Blues)	Online intervention, guided+TAU	Peer support specialist	TAU+depression workbook	In-person	VA^k^ physician
	Raevuori et al [34]	8	CBT (Meru Health Program)	Smartphone intervention, guided	Mental health specialist	TAU	In-person	Mental health specialist
	Wozney et al [35]	24	CBT (MOM: Managing Our Mood)	Handbook and telephone coaching	Trained coach	TAU	In-person	PCP
**KQ1c (virtual vs attention control)**								
	Flygare et al [36]	8	CBT	Online intervention, guided	Mental health specialist	Psychoed^l^	Online intervention, guided	Mental health specialist
	Johansson et al [37]^m^	10	Psychodynamic therapy	Online intervention, guided	Mental health specialist	Psychoed	Online intervention, guided	Mental health specialist
	Ly et al [38]^m^	8	BA	Smartphone, guided	Mental health specialist	Mindfulness	Smartphone, guided	Mental health specialist
	Oehler et al [39]	6	CBT (iFight Depression)	Online intervention, guided	Mental health specialist	Progressive muscle relaxation	Online intervention, guided	Mental health specialist
	Reins et al [40]	6	CBT (GET.ON Mood Enhancer)	Online intervention, guided	Mental health specialist	Psychoed	Online intervention, unguided	None
**KQ2 (in-person vs virtual)**								
	Andersson et al [41]	8	CBT	In-person group,8 sessions (60 min)	Mental health specialist	CBT	Online intervention, guided	Mental health specialist
	Egede et al [42]	8	BA	In-person, 8 sessions (60 min)	Mental health specialist	BA	Telemedicine (video), 8 sessions (60 min)	Mental health specialist
	Mohr et al [43]	18	CBT	In-person, 18 sessions (45 min)	Mental health specialist	CBT	Telemedicine (telephone), 18 sessions (45 min)	Mental health specialist
	Thase et al [13]	20	CBT	In-person, 20 sessions (50 min)	Mental health specialist	CBT (Good Days Ahead)	Online intervention, guided (Good Days Ahead)	Mental health specialist

^a^Each row represents an intervention arm. Some references are listed more than once because they provided data from multiple intervention arms.

^b^KQ: key question.

^c^CBT: cognitive behavioral therapy.

^d^NA: not applicable.

^e^ACT: acceptance and commitment therapy.

^f^BA: behavioral activation.

^g^TAU: treatment as usual.

^h^IPT: interpersonal therapy.

^i^OBGYN: obstetrician/gynecologist.

^j^PCP: primary care provider.

^k^VA: Veteran’s Administration.

^l^Psychoed: psychoeducation.

^m^Included in systematic review but excluded from meta-analysis due to differences in methods from other studies.

**Table 3:** Strength of evidence for each outcome organized by key question (KQ).

Outcome		Study design, duration, sample size (N), events^a^ (n)	Effect size (95% CI)	Factors that affect the strength of evidence	Overall evidence strength and direction of effect	Findings
**KQ1a (virtual vs waitlist)**						
	Remission	RCT^b^ (4 trials), 7-10 weeks, N=564, n=118 enrolled; N=619, n=126 analyzed due to 2 trials each having two intervention arms comparing to one control group	OR^c^ 10.30 (5.70 to 18.60)	Low ROB^d^, imprecise estimate^e^ but high effect (increase), direct consistent (*I*^2^= 0%)	High; virtual intervention>waitlist	The SOE^f^ is high that virtual interventions have 10 times higher odds of remission than waitlist
	Response	RCT (2 trials), 10-12 weeks, N=195, n=74 enrolled; N=221, n=78 analyzed due to 1 trial having two intervention arms comparing to the same control group	OR 3.57 (0.86 to 14.78)	1 Low, 1 moderate ROB (decrease),imprecise estimate (decrease), direct inconsistent^g^ (*I*^2^=79.1%) (decrease)	Low; no statistically significant difference	The SOE is low that there are no substantial differences in response between virtual interventions and waitlist
	Depression severity	RCT (7 trials), 7-12 weeks, N=1180, n=1180 enrolled; N=1071, n=1071 analyzed due to 2 trials each having two intervention arms comparing to one control group	SMD^h^ 0.81 (0.52 to 1.10)	4 Low, 3 moderate ROB; precise estimate; direct inconsistent (*I*^2^=77.8%) (decrease)	Moderate; virtual intervention>waitlist	The SOE is moderate that virtual interventions have greater reduction in depression severity compared with waitlist
**KQ1b (virtual vs TAU^i^)**						
	Remission	RCT (4 trials), 10-24 weeks; N=512, n=285	OR 2.27 (1.54 to 3.35)	Low ROB; imprecise estimate (decrease); direct consistent (*I*^2^=0%)	Moderate; virtual intervention>TAU	The SOE is moderate that virtual interventions have 2 times higher odds of remission than TAU
	Response	RCT (3 trials), 10-24 weeks; N=450, n=238	OR 2.95 (1.51 to 5.75)	Low ROB; imprecise estimate (decrease); direct consistent (*I*^2^= 58.0%)	Moderate; virtual intervention>TAU	The SOE is moderate that virtual interventions have 3 times higher odds of response than TAU
	Depression severity	RCT (7 trials), 8-24 weeks; N=1533, n=1533	SMD 0.59 (0.13 to 1.05)	5 Low, 2 Moderate ROB; precise estimate; direct inconsistent (*I*^2^=95.9%)	Moderate; virtual intervention>TAU	The SOE is moderate that virtual interventions have greater reduction in depression severity compared with TAU
**KQ1c (virtual vs attention control)**						
	Remission	RCT (2 trials), 6-8 weeks; N=226, n=99	OR 1.92 (1.10 to 3.35)	1 Low, 1 Moderate ROB (decrease); imprecise estimate (decrease); direct consistent (*I*^2^=50%)	Low; virtual CBT^j^>attention control	The SOE is low that virtual CBT has 2 times greater odds of remission than virtual psychoeducation
	Response	RCT (2 trials), 6-8 weeks; N=226, n=79	OR 1.68 (0.96 to 2.96)	1 Low, 1 Moderate ROB (decrease); imprecise estimate (decrease); direct consistent (*I*^2^=0%)	Low; no statistically significant difference	The SOE is low there are no substantial differences in response between virtual CBT and virtual psychoeducation
	Depression severity	RCT (3 trials), 6-8 weeks; N=573, n=573	SMD 0.25 (0.09 to 0.42)	2 Low, 1 Moderate ROB; precise estimate; direct consistent (*I*^2^=0%)	High; virtual CBT>attention control	The SOE is high that virtual CBT has greater reduction in depression severity compared with virtual psychoeducation
**KQ2 (in-person vs virtual)**						
	Remission	RCT (4 trials), 8-20 weeks; N=789, n=288,	OR 0.84 (0.51 to 1.37)	Low ROB; imprecise estimate (decrease); direct consistent (*I*^2^=60.1%)	Moderate; no statistically significant difference, noninferiority trials^f^	The SOE is moderate that there are no substantial differences in remission between in-person and virtual interventions
	Response	RCT (3 trials), 8-18 weeks; N=635, n=230	OR 0.82 (0.44 to 1.54)	Low ROB; imprecise estimate (decrease); direct consistent (*I*^2^=64.7%)	Moderate; no statistically significant difference, noninferiority trials	The SOE is moderate that there are no substantial differences in response between in-person and virtual interventions
	Depression severity	RCT (3 trials), 8-20 weeks; N=548, n=548	SMD –0.04 (–0.30 to 0.23)	Low ROB; precise estimate; direct consistent (*I*^2^=51.5%)	Moderate; no statistically significant difference, noninferiority trials	The SOE is moderate that there are no substantial differences in posttreatment depression severity between in-person and virtual interventions

^a^Based on risk of bias, precision of estimate, directness of comparison, and consistency.

^b^RCT: randomized controlled trial.

^c^OR: odds ratio.

^d^ROB: risk of bias.

^e^Imprecision is based on the number of events <300 events, or n=400 for continuous events or very wide confidence intervals; precision was the primary variable that influenced strength of evidence ratings given that most trials had low risk of bias and were direct and consistent.

^f^SOE: strength of evidence.

^g^Inconsistent was based on I^2^>75%.

^h^SMD: standardized mean difference.

^i^TAU: treatment as usual.

^j^CBT: cognitive behavioral therapy.

### Efficacy of Virtual Intervention Versus Waitlist Control (KQ1a)

The efficacy of virtual interventions compared with waitlist was assessed in seven double-blinded RCTs [11,23-28] (Table 2). Most trials compared virtual CBT with waitlist [11,23,24,26,28]: four trials examined virtual CBT guided by mental health providers [23,24,26,28], two examined unguided virtual CBT [11,24], and one examined CBT provided via email [24]. Two studies examined virtual adaptations of evidence-based therapies other than CBT (ie, combined acceptance and commitment therapy and behavioral activation [BA] [25] and problem-solving therapy [27]).

Remission was evaluated in four trials [11,23-25]. Two of the trials included comparisons of two different intervention arms against waitlist control groups: Berger et al [23] examined both guided and unguided virtual CBT compared with waitlist, and Vernmark et al [24] examined both guided virtual CBT and CBT provided via email compared with waitlist. Meta-analysis including a total of five comparisons across three studies showed that the odds of remission were 10 times higher (95% CI 5.70-18.60; N=619; high SOE) with virtual intervention compared with waitlist (Figure 2). Response was measured in three separate comparisons across two studies [23,28]. The odds of response did not substantially differ between virtual interventions and waitlist (OR 3.57, 95% CI 0.86-14.87; N=221; low SOE). Depression severity at posttreatment was assessed in seven trials [11,22–27]. Virtual interventions resulted in lower depression severity at posttreatment compared with waitlist (SMD 0.81, 95% CI 0.52-1.10; N=1071; moderate SOE).

### Efficacy of Virtual Intervention Versus TAU (KQ1b)

Efficacy of virtual interventions compared with TAU was evaluated in eight double-blinded RCTs [12,29-35] (Table 2). Three of the trials focused on interventions for specific populations: those with perinatal-onset MDD [12,29,35], a majority-male veteran cohort [33], and a Latinx Spanish-speaking population [32] (Table 1). Most virtual interventions involved guided virtual CBT [29-31,33-35]; however, two trials provided synchronous telehealth interventions, including interpersonal therapy delivered by nurses via telephone [12] and supportive therapy plus pharmacotherapy provided by a psychiatrist via video visits [32]. Most TAU study arms consisted of primary care appointments, scheduled on an as-needed basis delivered by physicians [29-33,35].

Remission was evaluated in four trials [12,29,32,35]. The odds of remission were two times higher with virtual intervention compared with TAU (OR 2.27, 95% CI 1.54-3.35; N=512; moderate SOE) (Figure 3). Response was evaluated in three trials [12,29,32]. The odds of response were nearly three times higher with virtual intervention compared with TAU (OR 2.95, 95% CI 1.51-5.75; N=450; moderate SOE). Depression severity was evaluated in seven trials [12,29-34]. Virtual intervention resulted in a lower depression severity at posttreatment compared with TAU (SMD 0.59, 95% CI 0.13-1.05; N=1533; moderate SOE).

### Efficacy of Virtual Therapy Versus Attention Control (KQ1c)

Five trials compared a virtual adaptation of an evidence-based intervention (eg, CBT [36,39,40], BA [38], or psychodynamic therapy [37]) with a virtual control (ie, mindfulness [38]) or sham condition (Table 1). Of these, three studies compared virtual CBT with attention control conditions, which included online psychoeducation [36,40] and progressive muscle relaxation [39]. These three studies were included in one set of meta-analyses based on the consistency in interventions (virtual CBT) and attention control conditions (Figure 4).

Remission and response were assessed in two trials [36,40], both of which compared virtual CBT to virtual psychoeducation and favored virtual CBT in terms of both remission and response (Figure 4). The odds of remission were higher with virtual CBT compared with virtual psychoeducation (OR 1.92, 95% CI 1.10-3.35; N=226; low SOE), whereas there was no statistically significant difference in response rates between virtual CBT and virtual psychoeducation (OR 1.68, 95% CI 0.96-2.96; N=226; low SOE). Depression severity at posttreatment was evaluated in three trials [36,39,40]. All three studies favored virtual CBT compared with an attention control condition. Virtual CBT resulted in lower depression severity at posttreatment compared with virtual psychoeducation (SMD 0.25, 95% CI 0.09-0.42; N=573; high SOE).

### Efficacy of In-Person Versus Virtual Intervention (KQ2)

Efficacy of in-person compared with virtual delivery of behavioral therapy, either CBT or BA, was evaluated in four RCTs [13,41-43] (Table 1), three of which were noninferiority trials [41-43] powered to evaluate whether virtual therapy provides at least the same benefit to the patient as in-person therapy. Two of the virtual interventions were guided virtual CBT [13,41] and two were synchronous telehealth interventions [42,43]. One trial compared in-person group CBT with virtual CBT [41].

Remission was evaluated in four trials [13,41,43]. In no study did remission rates for the in-person intervention exceed those seen in virtual interventions (Figure 5). Indeed, one trial reported significantly lower remission rates in the in-person CBT groups compared with virtual CBT (19% vs 52%; *P*<.005) [41]. The odds of remission with the in-person intervention were not higher than those with the virtual intervention (OR 0.84, 95% CI 0.51-1.37; N=789; moderate SOE). Sensitivity analysis excluding the study of Andersson et al [41], as the only trial comparing group therapy with virtual therapy, similarly indicated no significant difference but resolved the heterogeneity (OR 1.05, 95% CI 0.77-1.43; *I*^2^=0) (Multimedia Appendix 1, Figure A1).

Response was evaluated in three trials [41-43]. None of the trials reported better outcomes for the in-person arm. Indeed, after 8 weeks of intervention, in-person group CBT produced a significantly *lower* response rate than virtual CBT (25% vs 52%; *P*=.02) [41]. The odds of response with in-person intervention were no higher than those with virtual intervention (OR 0.82, 95% CI 0.44-1.54; N=635; moderate SOE) (Figure 5). Sensitivity analysis dropping the outlier [41] similarly indicated no statistically significant difference (Multimedia Appendix 1, Figure A1).

Depression severity was evaluated in all four trials [13,41-43]; none reported a benefit for in-person compared to virtual intervention. One trial comparing BA delivered in person versus via telehealth reported no statistically significant difference in depressive severity at posttreatment, but did not provide sufficient quantitative data [40] and was thus excluded from meta-analysis. In-person intervention was not associated with lower depression severity at posttreatment compared with virtual interventions (SMD –0.04, 95% CI –0.30 to 0.23; N=548; moderate SOE) (Figure 5) for the remaining three trials.

### Comparative Efficacy of Various Virtual Interventions (KQ3)

No trials comparing the efficacy of one virtual intervention with another virtual intervention were identified in our searches that met our inclusion criteria.

## Discussion

### Principal Results

Virtual intervention for individuals with mild to moderate depressive disorders resulted in higher remission rates and a lower severity of symptoms at posttreatment compared with waitlist, TAU, and attention control conditions. There was no consistent evidence that an in-person intervention is significantly more efficacious than a virtual intervention for depression. Two studies compared telehealth with in-person sessions, while two studies compared virtual behavior therapies with in-person sessions. Despite these methodological differences, heterogeneity between studies was low and sensitivity analyses showed no difference in results if any study was removed. Studies included individuals with mild to moderate depressive disorders across a number of patient populations, including primary care patients, veterans, perinatal women, and Spanish-speaking Latinx individuals, suggesting relatively broad generalizability to depressed populations in countries with a very high human development index. Of note, we found no eligible studies comparing the effectiveness of different active virtual interventions, which is an important research and clinical gap that should be addressed in future trials. Taken together, the results suggest that virtual therapy is an effective method of treatment for mild to moderate depressive disorders. The results further suggest a lack of clear evidence that in-person treatment is superior to virtual treatment for those with mild to moderate depressive disorders without significant comorbidity and living in countries with a very high human development index.

Given the significant limitations in access to evidence-based care in the United States, this represents a potential opportunity to increase access to effective and affordable treatment. Despite the finding that, on average, there is not reliable evidence that in-person treatment is superior to virtual treatment for depressive disorders, critical research to identify which patients benefit most from in-person and virtual treatment has not been done. Some patients may benefit more from in-person therapy than virtual treatment. Many people do not have access to high-speed internet, a private space for virtual sessions, or a home environment that is safe or conductive to engaging in therapy at home. None of the studies included in this review addressed these important individual differences that may differentially impact treatment feasibility, acceptability, and outcomes. As such, in-person therapy for mood disorders remains an important first-line treatment option. However, virtual therapy can be considered an additional first-line treatment option, particularly for those who prefer it and those without transportation, time, or geographical access to in-person treatment.

### Limitations

This systematic review and meta-analysis had important limitations. For some of the outcomes, a low number of events (ie, remission or response) observed across a small number of studies reduced the SOE, particularly in the case of response, which had the lowest number of observations of any outcome assessed and resulted in low to moderate SOE ratings across each key question. Our review was narrowly focused on depression intervention outcomes: all of the trials that met the inclusion criteria focused on interventions for MDD, and despite inclusion criteria of all depressive disorders, none examined other depressive disorders or other common co-occurring conditions such as anxiety disorders. Only one study included individuals with severe depressive symptoms, and therefore conclusions cannot be made regarding the utility of virtual therapy for those with more severe presentations. Interventions ranged in duration from 6 to 24 weeks. We evaluated immediate effects of the intervention on depression outcomes; however, due to variability in follow-up assessments, we did not examine long-term outcomes. Patient adherence to the intervention was not defined consistently across studies, and intervention fidelity was not assessed in most studies. Thus, neither variable could be evaluated as part of our risk of bias assessment. The studies included in KQ2 evaluated heterogeneous treatment populations (eg, veterans, primary care patients), and different in-person (eg, group therapy, individual therapy) and virtual (eg, telephone therapy, video therapy, virtual CBT) treatments. Although sensitivity analyses suggested that the results were the same when eliminating heterogeneous studies, additional studies are needed to have strong confidence in the results.

### Comparison With Prior Work

The results of this study were consistent with older meta-analyses establishing the efficacy of virtual CBT for depression and anxiety compared with no intervention [8-10], and with a recent meta-analysis examining the effectiveness [44] of open-label, nonrandomized virtual and other remote interventions for depression and anxiety, compared with control conditions. Similar to prior meta-analyses [10,45], we found that the effect size comparing virtual intervention with waitlist was larger than that for TAU. Our results were also similar to past meta-analyses showing that outcomes for virtual treatment were at least as good as outcomes for face-to-face therapy [45,46]. Our study extended these findings by including only individuals diagnosed with MDD and by examining not only depressive symptoms but also remission and response rates. To our knowledge, this was the first meta-analysis to compare virtual with face-to-face interventions for individuals with clinically confirmed diagnoses of depressive disorders with a focus on remission and response. Our review adds to the literature by (1) focusing on depressive disorders and not only depressive symptoms, which could be subthreshold, less severe, and less likely to show a difference between two interventions; and (2) including two noninferiority trials, which provides a stronger test of whether virtual therapy provides at least the same benefit as in-person therapy. With this more rigorous test, virtual interventions performed as well as face-to-face therapy.

### Strengths

Strengths of this systematic review included a multidimensional approach to assessing risk of bias, based on both the Cochrane risk of bias tool [20] and guidelines for applying the Cochrane tool to psychotherapy trials [21]. Psychotherapy trials, by definition, do not allow for participant blinding in the same way as medication trials. Yet, the underlying principle of blinding is believability of or confidence in the intervention to a similar degree across both the active intervention and control conditions. Only one trial with an active control condition assessed or attempted to control for participants’ confidence in the intervention. This absence represents a weakness in the psychotherapy literature that should be addressed by future trials. Other strengths of this systematic review were the inclusion of only RCTs, trials that required a depression diagnosis at baseline, use of a validated assessment of depression outcome, and those with low to medium risk of bias, which increased the strength of the conclusions.

### Conclusions

These results carry implications for health systems and mental health clinicians, policymakers, and researchers. Mental health clinics with long waitlists for evidence-based interventions and primary care clinics offering TAU could improve patient outcomes, reduce wait times, and reserve face-to-face sessions with therapists for those with the most severe symptoms by providing virtual interventions. With the rates of depression reaching epidemic proportions during the COVID-19 pandemic, existing efficacious technological solutions can help reduce the burden on the health care system, increase access to mental health care, and reduce the risk of COVID-19 transmission in health care settings. Implementation research is needed to determine when and for whom virtual interventions work best and when they may serve as an alternative to face-to-face therapy. Studies examining the efficacy of virtual adaptations of other evidence-based interventions for depression (eg, BA, acceptance and commitment therapy), optimal amount of guidance for virtual interventions (eg, regularly scheduled or as-needed coaching), optimal format for provider involvement (eg, telephone or email), and degree of provider training (eg, peer support, trained coaches, or licensed mental health providers) are needed to guide clinical decision-making. Nevertheless, our results suggest that virtual interventions provide an efficacious mechanism for scaling-up depression interventions to meet the growing demands created by the COVID-19 pandemic.

## Appendix

Multimedia Appendix 1 Supplementary information: Tables A1-A5; Figure A1.

## Abbreviations

AHRQ: Agency for Healthcare and Research Quality
BA: behavioral activation
CBT: cognitive behavioral therapy
KQ: key question
MDD: major depressive disorder
MeSH: Medical Subject Heading
OR: odds ratio
PRISMA: Preferred Reporting Items for Systematic Reviews and Meta-Analyses
RCT: randomized controlled trial
SMD: standardized mean difference
SOE: strength of evidence
TAU: treatment as usual
