# Supplementary material for: Efficacy of Virtual Care for Depressive Disorders: Systematic Review and Meta-analysis
Source: JMIR Ment Health. 2023 Jan 9;10:e38955. doi: 10.2196/38955 (PMC9871881; doi:10.2196/38955)
Supplement: Multimedia Appendix 1 [file mental_v10i1e38955_app1.doc]

## **Multimedia Appendix 1**

Table 1. Search Terms…………………………………………………………………………………………………………………….2

Table 2. Inclusion and exclusion criteria………………………………………………………………………………………………….3

Table 3. Countries with a very high human development index………………………………………………………………………….4

Table 4. Risk of bias assessment of included studies.… …………………………………………………………………………………5

Figure 1. Sensitivity analyses: In-Person treatment compared with virtual treatment (KQ2), excluding Andersson et al….……………6

Table 5. Strength of evidence grades and definitions based on AHRQ guidelines. …………………………………….………………..7

Table 6. Reasons for excluding studies from review….…………………………………………………………………………………..8

**Table 1. Search terms.**

| **Search** | **Query** |
| --- | --- |
| 1 | ("tele-medicine"[tiab] OR "tele medicine"[tiab] OR telemedicine[tiab] OR telehealth[tiab] OR "tele-health"[tiab] OR "tele health"[tiab] OR telecare[tiab] OR "tele-care"[tiab] OR "tele care"[tiab] OR telephone-based[tiab] OR telephonic[tiab] OR remote-consultation*[tiab] OR ehealth[tiab] OR mhealth[tiab] OR "e-health"[tiab] OR "m-health"[tiab] OR "mobile-health"[tiab] OR "mobile health"[tiab] OR "digital-health"[tw] OR "health-technology"[tiab] OR "digital health"[tw] OR "health technology"[tiab] OR digital[tiab] OR online[tiab] OR internet[tiab] OR website[tiab] OR websites[tiab] OR web-based[tiab] OR mobile-app[tiab] OR mobile-apps[tiab] OR app-based[tiab] OR mobile-application[tiab] OR mobile-applications[tiab] OR phone[tiab] OR phones[tiab] OR smartphone[tiab] OR text-message[tiab] OR text-messages[tiab] OR text-messaging[tiab] OR texting[tiab] OR wearable[tiab] OR wearables[tiab] OR Telemedicine[Majr] OR Internet Based Intervention[Majr]) |
| 2 | ((common-mental-disorder[tiab] OR common-mental-disorders[tiab] OR depression[tw] OR depressive-disorder[tiab] OR depressive-disorders[tiab] OR mood-disorder[tiab] OR mood-disorders[tiab] OR anxiety[tiab] OR generalized-anxiety-disorder[tw] OR acute-stress-disorder[tiab] OR acute-stress-disorders[tiab] OR traumatic-stress-disorder[tiab] OR traumatic-stress-disorders[tiab] OR post-traumatic-stress-disorder[tiab] OR posttraumatic-stress-disorder[tiab] OR post-traumatic-stress-disorders[tiab] OR posttraumatic-stress-disorders[tiab] OR PTSD[tiab] OR posttraumatic-neuroses[tiab] OR post-traumatic-neuroses[tiab] OR post-traumatic-neuroses[tiab]) AND (diagnosis[tiab] OR diagnose*[tiab] OR disorder[tiab] OR disorders[tiab] OR psychiatr*[tw] OR psychotherap*[tw] OR psycholog*[tw] OR cognitive-behavioral-therapy[tiab] OR cognitive-behavioural-therapy[tiab] OR clinical[tiab] or clinically[tiab] OR baseline[tiab] OR pre-existing[tiab])) OR (Depressive Disorder[Majr] OR Depressive Disorder, Major[Majr] OR Anxiety Disorders[Majr] OR Stress Disorders, Traumatic[Majr] OR Stress Disorders, Traumatic, Acute[Majr] OR Stress Disorders, Post Traumatic[Majr]) |
| 3 | (outcome[tiab] OR outcomes[tiab] OR patient-reported-outcome[tw] OR improve[tiab] OR improving[tiab] OR improvement[tiab] OR improvements[tiab] OR benefits[tiab] OR beneficial[tiab] OR increase[tiab] OR decrease[tiab] OR affect[tiab] OR affects[tiab] OR effective[tiab] OR effectiveness[tiab] OR efficacy[tiab] OR efficacies[tiab] OR Outcome Assessment, Health Care[Majr:NoExp] OR Patient Outcome Assessment[Majr:NoExp] OR Patient Reported Outcome Measures[Majr] OR Treatment Outcome[Majr:NoExp] OR Treatment Failure[Majr]) |
| 4 | 1 AND 2 AND 3 |
| 5 | 4 AND (clinical trials as topic[Mesh] OR clinical trial[pt] OR randomized controlled trial[pt] OR controlled clinical trial[pt] OR randomized[tiab] OR randomization[tiab] OR random-allocation[tiab] OR randomly[tiab] OR trial[tiab] OR groups[tiab] OR Control Groups[Mesh] OR Matched-Pair Analysis[Mesh] OR case-control[tiab] OR case-comparison[tiab] OR control-group[tiab] OR prospective[tiab] OR Prospective Studies[Mesh] OR cohort[tiab] or longitudinal[tiab]) AND English[Language] |
| 6 | 5 NOT (animals[Majr] NOT humans[Majr]) AND NOT (Neoplasms[Mesh] OR Surgical Procedures, Operative[Mesh] OR surgery[Subheading] OR cancer[ti] OR surgery[ti] OR Cross-Sectional Studies[Mesh] OR cross-sectional[ti] OR survey[ti] OR prevalence[ti] OR protocol[ti] OR meta analysis[Publication Type] OR systematic review[Publication Type] OR review[Publication Type] OR guideline[Publication Type] OR practice guideline[Publication Type] OR systematic-review[ti] OR meta-analysis[ti] OR scoping-review[ti] OR literature-review[ti]) |
| 7 | 6 AND "2005"[Date - Publication] : "2021"[Date - Publication] |
| 8 | 6 AND "2010"[Date - Publication] : "2021"[Date - Publication] |

**Table 2. Inclusion and exclusion criteria.**

|  | **Inclusion** | **Exclusion** | **Notes** |
| --- | --- | --- | --- |
| **Population** | Adults with a diagnosis of major depression, persistent depressive disorder, and dysthymic disorders | Not related to a cancer diagnosis or surgical/postoperative-focused | Adults: 18 years or older |
| **Intervention** | Treatments for depressive disorders that use virtual technologies | Complementary and alternative medicine approaches | e.g., telephone administered, virtual program (guided or unguided), virtual psychotherapy (individual or group), smartphone, email (messaging) therapy, telemedicine, telehealth, web-based |
| **Comparison condition** | **KQ1 :** Waitlist control, treatment as usual  **KQ2**: Virtual treatment  **KQ3:** head-to-head comparison with another virtual treatment | All other comparisons |  |
| **Outcomes** | Remission, response, and depression severity measured a validated scale (self-report or interviewer administered) |  | **Self-report to assess depressive symptoms**: BDI, CES-D, EPDS, GDS, PHQ-9, QIDS-SR16   **Interview-administered to assess depressive symptoms**: HAM-D / HRSD, MADRS, QIDS-CR16   **Interview-administered to assess for depressive diagnosis**:  MINI, SCID |
| **Time period** | At least 4 weeks study duration after randomization | Less than 4 weeks |  |
| **Setting** | Highly developed nations (see Table p. 4) | All other settings |  |
| **Designs** | Randomized controlled trials | All other designs and studies | Systematic reviews (for references), assess cluster randomized trials later |
| **Review Time Frame** | All papers published from 2010-present | Published before 2010 |  |
| **Sample Size** | ≥ 10 subjects | <10 subjects |  |
| **Language** | English | Studies published in languages other than English |  |

*Note.* BDI: Beck Depression Inventory; CES-D: Center for Epidemiological Study—Depression Scale; EPDS: Edinburgh Postnatal Depression Scale; GDS: Geriatric Depression Scale; HAM-D / HRSD: Hamilton Rating Scale for Depression; MADRS: Montgomery -Åsberg Depression Rating Scale; MINI: Mini International Neuropsychiatric Interview; PHQ-9: Patient Health Questionnaire; QIDS-CR16: Quick Inventory of Depressive Symptomatology; QIDS-SR16: Quick Inventory of Depressive Symptomatology; SCID: Structured Clinical Interview for the DSM.

**Table 3. Countries with a very high human development index. [19]**

| Andorra | Greece | Oman |
| --- | --- | --- |
| Argentina | Hong Kong China (SAR) | Palau |
| Australia | Hungary | Panama |
| Austria | Iceland | Poland |
| Bahamas | Ireland | Portugal |
| Bahrain | Israel | Qatar |
| Barbados | Italy | Romania |
| Belarus | Japan | Russian Federation |
| Belgium | Kazakhstan | Saudi Arabia |
| Brunei Darussalam | Korea (Republic of) | Serbia |
| Bulgaria | Latvia | Seychelles |
| Canada | Kuwait | Singapore |
| Chile | Liechtenstein | Slovakia |
| Croatia | Lithuania | Slovenia |
| Costa Rica | Luxembourg | Spain |
| Cyprus | Malaysia | Sweden |
| Czechia | Malta | Switzerland |
| Denmark | Mauritus | Turkey |
| Estonia | Montenegro | United Arab Emirates |
| Finland | Netherlands | United Kingdom |
| France | New Zealand | United States |
| Germany | Norway | Uroquay |

**Table 4. Risk of bias assessment of included studies.**

| **First author (year)** | **Random sequence generation (selection bias)** | **Allocation concealment (selection bias)** | **Groups similar at baseline** | **Assessed or controlled for patient expectations** | **Blinding of outcome assessment (detection bias)** | **Overall attrition <20% (attrition bias)** | **Differential attrition <15%** | **Study used ITT Analysis (reporting bias)** | **Overall Risk of Bias** |
| --- | --- | --- | --- | --- | --- | --- | --- | --- | --- |
| **KQ1a** |  |  |  |  |  |  |  |  |  |
| Berger (2011) | 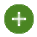 | 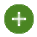 | 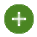 | 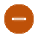 | 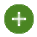 | 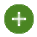 | 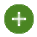 | 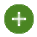 | 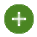 |
| Carlbring (2012) | 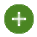 | 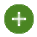 | 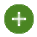 | 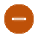 | 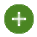 | 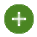 | 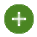 | 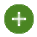 | 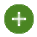 |
| Chan (2021) | 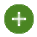 | 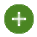 | 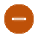 | 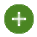 | 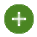 | 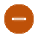 | 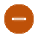 | 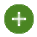 | 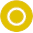 |
| Johansson (2019) | 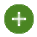 | 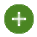 | 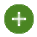 | 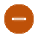 | 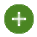 | 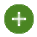 | 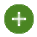 | 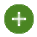 | 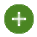 |
| Kenter (2016) | 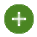 | 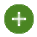 | 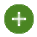 | 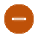 | 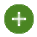 | 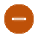 | 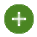 | 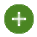 | 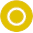 |
| Smith (2017) | 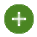 | 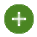 | 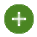 | 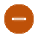 | 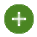 | 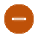 | 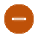 | 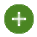 | 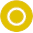 |
| Vernmark  (2010) | 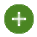 | 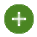 | 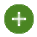 | 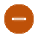 | 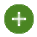 | 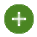 | 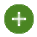 | 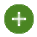 | 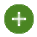 |
| **KQ1b** |  |  |  |  |  |  |  |  |  |
| Dennis (2020) | 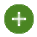 | 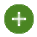 | 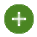 | 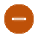 | 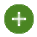 | 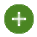 | 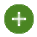 | 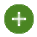 | 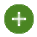 |
| Forsell (2017) | 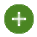 | 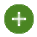 | 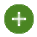 | 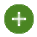 | 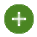 | 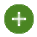 | 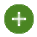 | 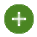 | 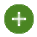 |
| Hallgren (2016) | 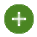 | 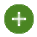 | 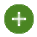 | 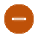 | 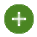 | 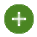 | 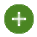 | 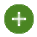 | 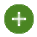 |
| Lobner (2018) | 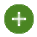 | 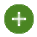 | 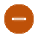 | 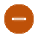 | 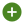 | 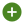 | 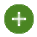 | 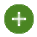 | 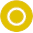 |
| Moreno (2012) | 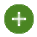 |  |  |  |  |  |  |  |  |
| Pfeiffer (2020) |  |  |  |  |  |  |  |  |  |
| Raevuori (2021) |  |  |  |  |  |  |  |  |  |
| Wozney (2017) |  |  |  |  |  |  |  |  |  |
| **KQ1c** |  |  |  |  |  |  |  |  |  |
| Flygare (2020) |  |  |  |  |  |  |  |  |  |
| Oehler (2020) |  |  |  |  |  |  |  |  |  |
| Reins (2018) |  |  |  |  |  |  |  |  |  |
| Johansson (2012)* |  |  |  |  |  |  |  |  |  |
| Ly (2014)* |  |  |  |  |  |  |  |  |  |
| **KQ2** |  |  |  |  |  |  |  |  |  |
| Andersson (2013) |  |  |  |  |  |  |  |  |  |
| Egede (2015) |  |  |  |  |  |  |  |  |  |
| Mohr (2012) |  |  |  |  |  |  |  |  |  |
| Thase (2018) |  |  |  |  |  |  |  |  |  |
| **Exlcuded Studies** |  |  |  |  |  |  |  |  |  |
| Abbott (2020) |  |  |  |  |  |  |  |  |  |
| Chong (2012) |  |  |  |  |  |  |  |  |  |
| Corruble (2016) |  |  |  |  |  |  |  |  |  |
| Eriksson (2017) |  |  |  |  |  |  |  |  |  |
| Gili (2020) |  |  |  |  |  |  |  |  |  |
| Haller (2018) |  |  |  |  |  |  |  |  |  |
| Hirsch (2018) |  |  |  |  |  |  |  |  |  |
| Høifødt (2013) |  |  |  |  |  |  |  |  |  |
| Montero-Marín (2016) |  |  |  |  |  |  |  |  |  |
| Pihlaja (2020) |  |  |  |  |  |  |  |  |  |
| Watts (2013) |  |  |  |  |  |  |  |  |  |

*Note. Based on the Cochrane Collaboration’s tool for assessing risk of bias: “+” indicates low risk of bias, “o” indicates some concerns, “-“ indicates high risk of bias, and “?” indicates no information. *=Included in systematic review but excluded from meta-analysis due to differences in methods from other studies.*

**Figure 1. Sensitivity analyses: In-Person treatment compared with virtual treatment (KQ2), excluding Andersson et al.**

***Remission***

***Response***

***Depression severity at post-treatment***

**Table 5. Strength of evidence grades and definitions.**

| **Grade** | **Definition** |
| --- | --- |
| **High** | **We are very confident that the estimate of effect lies close to the true effect for this outcome**. The body of evidence has few or no deficiencies. We believe that the findings are stable, i.e., another study would not change the conclusions. |
| **Moderate** | **We are moderately confident that the estimate of effect lies close to the true effect for this outcome.** The body of evidence has some deficiencies. We believe that the findings are likely to be stable, but some doubt remains. |
| **Low** | **We have limited confidence that the estimate of effect lies close to the true effect for this outcome.** The body of evidence has major or numerous deficiencies (or both). We believe that additional evidence is needed before concluding either that the findings are stable or that the estimate of effect is close to the true effect. |
| **Insufficient** | **We have no evidence, we are unable to estimate an effect, or we have no confidence in the estimate of effect for this outcome.** Either no evidence is available, or the body of evidence has unacceptable deficiencies, precluding reaching a conclusion. |

*Note:* Based on AHRQ guidelines [16].

**Table 6. Reasons for excluding studies from review.**

| Exclusion reason | Citation |
| --- | --- |
| High Risk of Bias | Abbott RD, Sherwin K, Klopf H, Mattingly HJ, Brogan K. Efficacy of a Multimodal Online Lifestyle Intervention for Depressive Symptoms and Quality of Life in Individuals With a History of Major Depressive Disorder. Cureus 2020; 12: e9061. |
| Wrong patient population | Acierno R, Knapp R, Tuerk P, et al. A non-inferiority trial of Prolonged Exposure for posttraumatic stress disorder: In person versus home-based telehealth. Behav Res Ther 2017; 89: 57-65. |
| Wrong patient population | Acierno Ron, Gros Daniel F, Ruggiero Kenneth J, et al. Behavioral activation and therapeutic exposure for posttraumatic stress disorder: A noninferiority trial of treatment delivered in person versus home‚Äêbased telehealth. Depression and Anxiety 2016; 33: 415-423. |
| Wrong indication | Adler DA, Lerner D, Visco ZL, et al. Improving work outcomes of dysthymia (persistent depressive disorder) in an employed population. Gen Hosp Psychiatry 2015; 37: 352-9. |
| Wrong intervention | Agyapong VI, Ahern S, McLoughlin DM, Farren CK. Supportive text messaging for depression and comorbid alcohol use disorder: single-blind randomised trial. J Affect Disord 2012; 141: 168-76. |
| Wrong patient population | Agyapong VIO, Juhas M, Ohinmaa A, et al. Randomized controlled pilot trial of supportive text messages for patients with depression. BMC Psychiatry 2017; 17: 286. |
| Wrong intervention | Aikens JE, Trivedi R, Heapy A, Pfeiffer PN, Piette JD. Potential Impact of Incorporating a Patient-Selected Support Person into mHealth for Depression. J Gen Intern Med 2015; 30: 797-803. |
| Wrong indication | Al-Asadi AM, Klein B, Meyer D. Posttreatment attrition and its predictors, attrition bias, and treatment efficacy of the anxiety online programs. J Med Internet Res 2014; 16: e232. |
| Wrong patient population | Al-Refae M, Al-Refae A, Munroe M, Sardella NA, Ferrari M. A Self-Compassion and Mindfulness-Based Cognitive Mobile Intervention (Serene) for Depression, Anxiety, and Stress: Promoting Adaptive Emotional Regulation and Wisdom. Front Psychol 2021; 12: 648087. |
| Wrong patient population | Alavi N, Hirji A, Sutton C, Naeem F. Online CBT Is Effective in Overcoming Cultural and Language Barriers in Patients With Depression. J Psychiatr Pract 2016; 22: 44600. |
| Wrong patient population | Alavi N, Hirji A. The Efficacy of PowerPoint-based CBT Delivered Through Email: Breaking the Barriers to Treatment for Generalized Anxiety Disorder. J Psychiatr Pract 2020; 26: 89-100. |
| Study Design Only - No Results | Allen AR, Newby JM, Smith J, Andrews G. Internet-based cognitive behavioural therapy (iCBT) for posttraumatic stress disorder versus waitlist control: Study protocol for a randomised controlled trial. Trials 2015; 16: . |
| Wrong patient population | Amir Nader, Taboas William, Montero Miguel. Feasibility and dissemination of a computerized home-based treatment for generalized anxiety disorder: A randomized clinical trial. Behaviour Research and Therapy 2019; 120: . |
| Duplicate Study | Andersson G, Hesser H, Hummerdal D, Bergman-Nordgren L, Carlbring P. A 3.5-year follow-up of Internet-delivered cognitive behavior therapy for major depression. J Ment Health 2013; 22: 155-64. |
| Wrong patient population | Andersson G, Paxling B, Roch-Norlund P, et al. Internet-based psychodynamic versus cognitive behavioral guided self-help for generalized anxiety disorder: a randomized controlled trial. Psychother Psychosom 2012; 81: 344-55. |
| Wrong patient population | Arean PA, Hallgren KA, Jordan JT, Gazzaley A, Atkins DC, Heagerty PJ, Anguera JA. The Use and Effectiveness of Mobile Apps for Depression: Results From a Fully Remote Clinical Trial. J Med Internet Res 2016; 18: e330. |
| Wrong patient population | Arjadi R, Nauta MH, Scholte WF, et al. Internet-based behavioural activation with lay counsellor support versus online minimal psychoeducation without support for treatment of depression: a randomised controlled trial in Indonesia. Lancet Psychiatry 2018; 5: 707-716. |
| Wrong intervention | Baggett KM, Davis B, Sheeber LB, Ammerman RT, Mosley EA, Miller K, Feil EG. Minding the Gatekeepers: Referral and Recruitment of Postpartum Mothers with Depression into a Randomized Controlled Trial of a Mobile Internet Parenting Intervention to Improve Mood and Optimize Infant Social Communication Outcomes. Int J Environ Res Public Health 2020; 17: . |
| Wrong patient population | Bakker D, Kazantzis N, Rickwood D, Rickard N. A randomized controlled trial of three smartphone apps for enhancing public mental health. Behav Res Ther 2018; 109: 75-83. |
| Wrong intervention | Balestrieri M, Sisti D, Rocchi M, Rucci P, Simon G, Araya R, de Girolamo G. Effectiveness of clinical decision support systems and telemedicine on outcomes of depression: a cluster randomized trial in general practice. Fam Pract 2020; : . |
| Wrong patient population | Barkham M, Saxon D, Hardy GE, et al.. Person-centred experiential therapy versus cognitive behavioural therapy delivered in the English Improving Access to Psychological Therapies service for the treatment of moderate or severe depression (PRaCTICED): a pragmatic, randomised, non-inferiority . Lancet Psychiatry 2021; 8: 487-499. |
| Wrong study design | Bastiaansen JA, Orn√©e DA, Meurs M, Oldehinkel AJ. An evaluation of the efficacy of two add-on ecological momentary intervention modules for depression in a pragmatic randomized controlled trial (ZELF-i). Psychol Med 2020; : 44571. |
| Wrong intervention | Batterham PJ, Calear AL, Sunderland M, et al.. A Brief Intervention to Increase Uptake and Adherence of an Internet-Based Program for Depression and Anxiety (Enhancing Engagement With Psychosocial Interventions): Randomized Controlled Trial. J Med Internet Res 2021; 23: e23029. |
| Wrong study design | Baumel A, Tinkelman A, Mathur N, Kane JM. Digital Peer-Support Platform (7Cups) as an Adjunct Treatment for Women With Postpartum Depression: Feasibility, Acceptability, and Preliminary Efficacy Study. JMIR Mhealth Uhealth 2018; 6: e38. |
| Wrong patient population | Bee PE, Bower P, Gilbody S, Lovell K. Improving health and productivity of depressed workers: a pilot randomized controlled trial of telephone cognitive behavioral therapy delivery in workplace settings. Gen Hosp Psychiatry 2010; 32: 337-40. |
| Wrong patient population | Beevers CG, Pearson R, Hoffman JS, Foulser AA, Shumake J, Meyer B. Effectiveness of an internet intervention (Deprexis) for depression in a united states adult sample: A parallel-group pragmatic randomized controlled trial. J Consult Clin Psychol 2017; 85: 367-380. |
| Wrong patient population | Beiwinkel T, Eissing T, Telle NT, Siegmund-Schultze E, Rossler W. Effectiveness of a Web-Based Intervention in Reducing Depression and Sickness Absence: Randomized Controlled Trial. J Med Internet Res 2017; 19: e213. |
| Wrong patient population | Ben-Zeev D, Brian RM, Jonathan G, et al. Mobile Health (mHealth) Versus Clinic-Based Group Intervention for People With Serious Mental Illness: A Randomized Controlled Trial. Psychiatr Serv 2018; 69: 978-985. |
| Wrong study design | Berger T, Krieger T, Sude K, Meyer B, Maercker A. Evaluating an e-mental health program ("deprexis") as adjunctive treatment tool in psychotherapy for depression: Results of a pragmatic randomized controlled trial. J Affect Disord 2018; 227: 455-462. |
| Wrong patient population | Berger T, Urech A, Krieger T, et al. Effects of a transdiagnostic unguided Internet intervention ('velibra') for anxiety disorders in primary care: results of a randomized controlled trial. Psychol Med 2017; 47: 67-80. |
| Wrong patient population | Berger Thomas, Boettcher Johanna, Caspar Franz. Internet-based guided self-help for several anxiety disorders: A randomized controlled trial comparing a tailored with a standardized disorder-specific approach. Psychotherapy 2014; 51: 207-219. |
| Study Design Only - No Results | Bergman Nordgren L, Andersson G, Kadowaki A, Carlbring P. Tailored internet-administered treatment of anxiety disorders for primary care patients: Study protocol for a randomised controlled trial. Trials 2012; : 16. |
| Wrong patient population | Biesheuvel-Leliefeld KEM, Dijkstra-Kersten SMA, van Schaik DJF, van Marwijk HWJ, Smit F, van der Horst HE, Bockting CLH. Effectiveness of Supported Self-Help in Recurrent Depression: A Randomized Controlled Trial in Primary Care. Psychother Psychosom 2017; 86: 220-230. |
| Wrong patient population | Birney AJ, Gunn R, Russell JK, Ary DV. MoodHacker Mobile Web App With Email for Adults to Self-Manage Mild-to-Moderate Depression: Randomized Controlled Trial. JMIR Mhealth Uhealth 2016; 4: e8. |
| Wrong patient population | Boersma K, Sodermark M, Hesser H, Flink IK, Gerdle B, Linton SJ. Efficacy of a transdiagnostic emotion-focused exposure treatment for chronic pain patients with comorbid anxiety and depression: a randomized controlled trial. Pain 2019; 160: 1708-1718. |
| Wrong patient population | Boeschoten RE, Dekker J, Uitdehaag BM, et al. Internet-based treatment for depression in multiple sclerosis: A randomized controlled trial. Mult Scler 2017; 23: 1112-1122. |
| Wrong patient population | Boettcher J, Astrom V, Pahlsson D, Schenstrom O, Andersson G, Carlbring P. Internet-based mindfulness treatment for anxiety disorders: a randomized controlled trial. Behav Ther 2014; 45: 241-53. |
| Wrong patient population | Boettcher J, Hasselrot J, Sund E, Andersson G, Carlbring P. Combining attention training with internet-based cognitive-behavioural self-help for social anxiety: a randomised controlled trial. Cogn Behav Ther 2014; 43: 34-48. |
| Wrong patient population | Boettcher Johanna, Magnusson Kristoffer, Marklund Arvid, et al. Adding a smartphone app to internet-based self-help for social anxiety: A randomized controlled trial. Computers in Human Behavior 2018; 87: 98-108. |
| Wrong study design - qualitative study | Boggs JM, Beck A, Felder JN, Dimidjian S, Metcalf CA, Segal ZV. Web-based intervention in mindfulness meditation for reducing residual depressive symptoms and relapse prophylaxis: a qualitative study. J Med Internet Res 2014; 16: e87. |
| Study Design Only - No Results | Bolier L, Haverman M, Kramer J, Boon B, Smit F, Riper H, Bohlmeijer E. Internet-based intervention to promote mental fitness in mildly depressed adults: design of a randomized controlled trial. JMIR Res Protoc 2012; 1: e2. |
| Wrong patient population | Bombardier CH, Ehde DM, Gibbons LE, Wadhwani R, Sullivan MD, Rosenberg DE, Kraft GH. Telephone-based physical activity counseling for major depression in people with multiple sclerosis. J Consult Clin Psychol 2013; 81: 89-99. |
| Wrong patient population | Boschloo L, Cuijpers P, Karyotaki E, Berger T, Moritz S, Meyer B, Klein JP. Symptom-specific effectiveness of an internet-based intervention in the treatment of mild to moderate depressive symptomatology: The potential of network estimation techniques. Behav Res Ther 2019; 122: 103440. |
| Wrong study design | Bottche M, Kuwert P, Pietrzak RH, Knaevelsrud C. Predictors of outcome of an Internet-based cognitive-behavioural therapy for post-traumatic stress disorder in older adults. Psychol Psychother 2016; 89: 82-96. |
| Wrong study design | Boykin DM, Keegan F, Thompson KE, Voelkel E, Lindsay JA, Fletcher TL. Video to Home Delivery of Evidence-Based Psychotherapy to Veterans With Posttraumatic Stress Disorder. Front Psychiatry 2019; 10: 893. |
| Wrong patient population | Brabyn S, Araya R, Barkham M, et al. The second Randomised Evaluation of the Effectiveness, cost-effectiveness and Acceptability of Computerised Therapy (REEACT-2) trial: does the provision of telephone support enhance the effectiveness of computer-delivered cognitive behaviour therapy? A ra. Health Technol Assess 2016; 20: 23377. |
| Wrong patient population | Braun L, Titzler I, Terhorst Y, Freund J, Thielecke J, Ebert DD, Baumeister H. Effectiveness of guided internet-based interventions in the indicated prevention of depression in green professions (PROD-A): Results of a pragmatic randomized controlled trial. J Affect Disord 2021; 278: 658-671. |
| Wrong patient population | Brenes GA, Danhauer SC, Lyles MF, Anderson A, Miller ME. Long-Term Effects of Telephone-Delivered Psychotherapy for Late-Life GAD. American Journal of Geriatric Psychiatry 2017; 25: 1249-1257. |
| Wrong patient population | Brenes GA, Danhauer SC, Lyles MF, Hogan PE, Miller ME. Telephone-Delivered Cognitive Behavioral Therapy and Telephone-Delivered Nondirective Supportive Therapy for Rural Older Adults With Generalized Anxiety Disorder: A Randomized Clinical Trial. JAMA Psychiatry 2015; 72: 1012-20. |
| Wrong patient population | Brenes GA. Telephone-delivered psychotherapy for late-life anxiety. Psychological Services 2012; 9: 219-220. |
| Wrong patient population | Brief DJ, Rubin A, Keane TM, et al. Web intervention for OEF/OIF veterans with problem drinking and PTSD symptoms: a randomized clinical trial. J Consult Clin Psychol 2013; 81: 890-900. |
| Wrong intervention | Broglia E, Millings A, Barkham M. Counseling With Guided Use of a Mobile Well-Being App for Students Experiencing Anxiety or Depression: Clinical Outcomes of a Feasibility Trial Embedded in a Student Counseling Service. JMIR Mhealth Uhealth 2019; 7: e14318. |
| Wrong patient population | Bucker L, Schnakenberg P, Karyotaki E, Moritz S, Westermann S. Diminishing Effects After Recurrent Use of Self-Guided Internet-Based Interventions in Depression: Randomized Controlled Trial. J Med Internet Res 2019; 21: e14240. |
| Wrong patient population | Buhrman M, Syk M, Burvall O, Hartig T, Gordh T, Andersson G. Individualized Guided Internet-delivered Cognitive-Behavior Therapy for Chronic Pain Patients With Comorbid Depression and Anxiety: A Randomized Controlled Trial. Clin J Pain 2015; 31: 504-16. |
| Wrong study design | Burns MN, Begale M, Duffecy J, Gergle D, Karr CJ, Giangrande E, Mohr DC. Harnessing context sensing to develop a mobile intervention for depression. J Med Internet Res 2011; 13: e55. |
| Wrong patient population | Butler Emma, Mobini Sirous, Rapee Ronald M, Mackintosh Bundy, Reynolds Shirley A. Enhanced effects of combined cognitive bias modification and computerised cognitive behaviour therapy on social anxiety. Cogent Psychology 2015; 2: . |
| Wrong study design | Button KS, Wiles NJ, Lewis G, Peters TJ, Kessler D. Factors associated with differential response to online cognitive behavioural therapy. Soc Psychiatry Psychiatr Epidemiol 2012; 47: 827-33. |
| Wrong patient population | Byatt N, Moore Simas TA, Biebel K, et al. PRogram In Support of Moms (PRISM): a pilot group randomized controlled trial of two approaches to improving depression among perinatal women. J Psychosom Obstet Gynaecol 2018; 39: 297-306. |
| Wrong patient population | Carlbring Per, Maurin Linda, T√∂rngren Charlotta, et al. Individually-tailored, internet-based treatment for anxiety disorders: A randomized controlled trial. Behaviour Research and Therapy 2011; 49: 18-24. |
| Wrong indication | Carter Frances A, Bell Caroline J, Colhoun Helen C. Suitability and acceptability of computerised cognitive behaviour therapy for anxiety disorders in secondary care. Australian and New Zealand Journal of Psychiatry 2013; 47: 142-152. |
| Wrong patient population | Cartreine JA, Locke SE, Buckey JC, Sandoval L, Hegel MT. Electronic problem-solving treatment: description and pilot study of an interactive media treatment for depression. JMIR Res Protoc 2012; 1: e11. |
| Wrong study design | Castro A, Garc√≠a-Palacios A, L√≥pez-Del-Hoyo Y, et al. Predictors of Adherence in Three Low-Intensity Intervention Programs Applied by ICTs for Depression in Primary Care. Int J Environ Res Public Health 2021; 18: . |
| Wrong indication | Castro A, Lopez-Del-Hoyo Y, Peake C, et al. Adherence predictors in an Internet-based Intervention program for depression. Cogn Behav Ther 2018; 47: 246-261. |
| Study Design Only - No Results | Castro Adoraci√≥n, Garc√≠a-Palacios Azucena, Garc√≠a-Campayo Javier, et al. Efficacy of low-intensity psychological intervention applied by ICTs for the treatment of depression in primary care: A controlled trial. BMC Psychiatry 2015; 15: . |
| Wrong patient population | Chen Huijing, Rodriguez Marcus A, Qian Mingyi, Kishimoto Tomoko, Lin Muyu, Berger Thomas. Predictors of treatment outcomes and adherence in internet-based cognitive behavioral therapy for social anxiety in china. Behavioural and Cognitive Psychotherapy 2020; : . |
| Wrong setting | Chen YL, Pan AW, Hsiung PC, Chung L, Lai JS, Shur-Fen Gau S, Chen TJ. Life Adaptation Skills Training (LAST) for persons with depression: A randomized controlled study. J Affect Disord 2015; 185: 108-14. |
| Wrong indication | Chen YL, Pan AW, Hsiung PC, Chung L. Quality of life enhancement programme for individuals with mood disorder: A randomized controlled pilot study. Hong Kong Journal of Occupational Therapy 2015; 25: 23-31. |
| Wrong patient population | Cho CH, Lee T, Lee JB, et al. Effectiveness of a Smartphone App With a Wearable Activity Tracker in Preventing the Recurrence of Mood Disorders: Prospective Case-Control Study. JMIR Ment Health 2020; 7: e21283. |
| Wrong patient population | Choi NG, Marti CN, Bruce ML, Hegel MT, Wilson NL, Kunik ME. Six-month postintervention depression and disability outcomes of in-home telehealth problem-solving therapy for depressed, low-income homebound older adults. Depress Anxiety 2014; 31: 653-61. |
| High Risk of Bias | Chong J, Moreno F. Feasibility and acceptability of clinic-based telepsychiatry for low-income Hispanic primary care patients. Telemed J E Health 2012; 18: 297-304. |
| Not original research | Christensen H, Batterham P, Calear A. Online interventions for anxiety disorders. Current Opinion in Psychiatry 2013; : . |
| Wrong study design | Christensen H, Batterham P, Mackinnon A, et al. Prevention of generalized anxiety disorder using a web intervention, iChill: randomized controlled trial. J Med Internet Res 2014; 16: e199. |
| Wrong patient population | Christensen H, Mackinnon AJ, Batterham PJ, et al. The effectiveness of an online e-health application compared to attention placebo or Sertraline in the treatment of Generalised Anxiety Disorder. Internet Interventions 2014; 1: 169-174. |
| Wrong patient population | Christoforou M, Saez Fonseca JA, Tsakanikos E. Two Novel Cognitive Behavioral Therapy-Based Mobile Apps for Agoraphobia: Randomized Controlled Trial. J Med Internet Res 2017; 19: e398. |
| Wrong patient population | Collins Sarah, Byrne Michael, Hawe James, O'Reilly Gary. Evaluation of a computerized cognitive behavioural therapy programme, mindwise (20), for adults with mild‚Äêto‚Äêmoderate depression and anxiety. British Journal of Clinical Psychology 2018; 57: 255-269. |
| High Risk of Bias | Corruble E, Swartz HA, Bottai T, et al. Telephone-administered psychotherapy in combination with antidepressant medication for the acute treatment of major depressive disorder. Journal of Affective Disorders 2016; 190: 44723. |
| Wrong patient population | Cougle JR, Wilver NL, Day TN, Summers BJ, Okey SA, Carlton CN. Interpretation Bias Modification Versus Progressive Muscle Relaxation for Social Anxiety Disorder: A Web-Based Controlled Trial. Behav Ther 2020; 51: 99-112. |
| Wrong patient population | Dagoo J, Asplund RP, Bsenko HA, et al. Cognitive behavior therapy versus interpersonal psychotherapy for social anxiety disorder delivered via smartphone and computer: a randomized controlled trial. J Anxiety Disord 2014; 28: 410-7. |
| Wrong patient population | Dahlin M, Andersson G, Magnusson K, et al. Internet-delivered acceptance-based behaviour therapy for generalized anxiety disorder: A randomized controlled trial. Behav Res Ther 2016; 77: 86-95. |
| Wrong patient population | Dahne J, Collado A, Lejuez CW, et al. Pilot randomized controlled trial of a Spanish-language Behavioral Activation mobile app ( inverted exclamation markAptivate!) for the treatment of depressive symptoms among united states Latinx adults with limited English proficiency. J Affect Disord 2019; 250: 210-217. |
| Wrong patient population | Dahne J, Lejuez CW, Diaz VA, Player MS, Kustanowitz J, Felton JW, Carpenter MJ. Pilot Randomized Trial of a Self-Help Behavioral Activation Mobile App for Utilization in Primary Care. Behav Ther 2019; 50: 817-827. |
| Wrong patient population | Day V, McGrath PJ, Wojtowicz M. Internet-based guided self-help for university students with anxiety, depression and stress: a randomized controlled clinical trial. Behav Res Ther 2013; 51: 344-51. |
| Non English | de Graaf Esther, Gerhards Sylvia, Arntz Arnoud, et al. Effectiviteit van online cognitieve gedragstherapie voor depressie in de eerste lijn = Effectiveness of online cognitive behaviour therapy for depression in primary care. Gedragstherapie 2010; 43: 59-77. |
| Wrong patient population | de Graaf LE, Hollon SD, Huibers MJ. Predicting outcome in computerized cognitive behavioral therapy for depression in primary care: A randomized trial. J Consult Clin Psychol 2010; 78: 184-9. |
| Wrong patient population | De Jaegere E, van Landschoot R, van Heeringen K, van Spijker BAJ, Kerkhof Ajfm, Mokkenstorm JK, Portzky G. The online treatment of suicidal ideation: A randomised controlled trial of an unguided web-based intervention. Behav Res Ther 2019; 119: 103406. |
| Wrong patient population | Deady M, Glozier N, Collins D, et al. The Utility of a Mental Health App in Apprentice Workers: A Pilot Study. Front Public Health 2020; 8: 389. |
| Wrong patient population | Deady M, Mills KL, Teesson M, Kay-Lambkin F. An Online Intervention for Co-Occurring Depression and Problematic Alcohol Use in Young People: Primary Outcomes From a Randomized Controlled Trial. J Med Internet Res 2016; 18: e71. |
| Wrong patient population | Dear BF, Fogliati VJ, Fogliati R, et al. Treating anxiety and depression in young adults: A randomised controlled trial comparing clinician-guided versus self-guided Internet-delivered cognitive behavioural therapy. Aust N Z J Psychiatry 2018; 52: 668-679. |
| Wrong patient population | Dear BF, Johnson B, Singh A, et al. Examining an internet-delivered intervention for anxiety and depression when delivered as a part of routine care for university students: A phase IV trial. J Affect Disord 2019; 256: 567-577. |
| Wrong patient population | Dear BF, Staples LG, Terides MD, et al. Transdiagnostic versus disorder-specific and clinician-guided versus self-guided internet-delivered treatment for Social Anxiety Disorder and comorbid disorders: A randomized controlled trial. Journal of Anxiety Disorders 2016; 42: 30-44. |
| Wrong study design | Dear BF, Titov N, Schwencke G, Andrews G, Johnston L, Craske MG, McEvoy P. An open trial of a brief transdiagnostic internet treatment for anxiety and depression. Behav Res Ther 2011; 49: 830-7. |
| Wrong study design | Dear BF, Zou J, Titov N, et al. Internet-delivered cognitive behavioural therapy for depression: a feasibility open trial for older adults. Aust N Z J Psychiatry 2013; 47: 169-76. |
| Wrong intervention | Delsignore A, Rufer M, Emmerich J, Weidt S, Bruhl AB, Moergeli H. E-mail support as an adjunct to cognitive-behavioral group therapy for social anxiety disorder: Impact on dropout and outcome. Psychiatry Res 2016; 244: 151-8. |
| Wrong patient population | Dimidjian S, Beck A, Felder JN, Boggs JM, Gallop R, Segal ZV. Web-based Mindfulness-based Cognitive Therapy for reducing residual depressive symptoms: An open trial and quasi-experimental comparison to propensity score matched controls. Behav Res Ther 2014; 63: 83-9. |
| Wrong study design | Doukani A, Free C, Michelson D, et al. Towards a conceptual framework of the working alliance in a blended low-intensity cognitive behavioural therapy intervention for depression in primary mental health care: a qualitative study. BMJ Open 2020; 10: e036299. |
| Wrong patient population | Douma M, Maurice-Stam H, Gorter B, et al. Online psychosocial group intervention for parents: Positive effects on anxiety and depression. J Pediatr Psychol 2021; 46: 123-134. |
| Wrong patient population | Dwight-Johnson M, Aisenberg E, Golinelli D, Hong S, O'Brien M, Ludman E. Telephone-based cognitive-behavioral therapy for Latino patients living in rural areas: a randomized pilot study. Psychiatr Serv 2011; 62: 936-42. |
| Wrong patient population | Ebert DD, Lehr D, Boss L, et al. Efficacy of an internet-based problem-solving training for teachers: results of a randomized controlled trial. Scand J Work Environ Health 2014; 40: 582-96. |
| Wrong patient population | Ebert DD, Nobis S, Lehr D, et al. The 6-month effectiveness of Internet-based guided self-help for depression in adults with Type 1 and 2 diabetes mellitus. Diabet Med 2017; 34: 99-107. |
| Wrong patient population | Egede LE, Acierno R, Knapp RG, Walker RJ, Payne EH, Frueh BC. Psychotherapy for Depression in Older Veterans Via Telemedicine: Effect on Quality of Life, Satisfaction, Treatment Credibility, and Service Delivery Perception. J Clin Psychiatry 2016; 77: 1704-1711. |
| Wrong patient population | El Alaoui S, Hedman E, Ljotsson B, Lindefors N. Long-term effectiveness and outcome predictors of therapist-guided internet-based cognitive-behavioural therapy for social anxiety disorder in routine psychiatric care. BMJ Open 2015; 5: e007902. |
| Wrong study design | El Alaoui Samir, Hedman Erik, Kaldo Viktor, et al. Effectiveness of Internet-based cognitive‚Äìbehavior therapy for social anxiety disorder in clinical psychiatry. Journal of Consulting and Clinical Psychology 2015; 83: 902-914. |
| Wrong patient population | El Morr C, Ritvo P, Ahmad F, Moineddin R. Effectiveness of an 8-Week Web-Based Mindfulness Virtual Community Intervention for University Students on Symptoms of Stress, Anxiety, and Depression: Randomized Controlled Trial. JMIR Ment Health 2020; 7: e18595. |
| Wrong patient population | Elison S, Ward J, Williams C, et al. Feasibility of a UK community-based, eTherapy mental health service in Greater Manchester: repeated-measures and between-groups study of 'Living Life to the Full Interactive', 'Sleepio' and 'Breaking Free Online' at 'Self Help Services'. BMJ Open 2017; 7: e016392. |
| Wrong patient population | Ellis Louise A, Campbell Andrew J, Sethi Suvena, O'Dea Bridianne M. Comparative randomized trial of an online cognitive-behavioral therapy program and an online support group for depression and anxiety. Journal of Cybertherapy and Rehabilitation 2011; 4: 461-467. |
| Wrong patient population | Engel CC, Jaycox LH, Freed MC, et al. Centrally Assisted Collaborative Telecare for Posttraumatic Stress Disorder and Depression Among Military Personnel Attending Primary Care: A Randomized Clinical Trial. JAMA Intern Med 2016; 176: 948-56. |
| Wrong patient population | Engel CC, Litz B, Magruder KM, et al. Delivery of self training and education for stressful situations (DESTRESS-PC): a randomized trial of nurse assisted online self-management for PTSD in primary care. Gen Hosp Psychiatry 2015; 37: 323-8. |
| Wrong outcomes | Enrique A, Burke J, Richards D, Timulak L. Quality of life outcomes in internet-delivered (space from depression) treatment for depression. Applied Research in Quality of Life 2018; : . |
| Wrong patient population | Enrique A, Palacios JE, Ryan H, Richards D. Exploring the Relationship Between Usage and Outcomes of an Internet-Based Intervention for Individuals With Depressive Symptoms: Secondary Analysis of Data From a Randomized Controlled Trial. J Med Internet Res 2019; 21: e12775. |
| High Risk of Bias | Eriksson MCM, Kivi M, Hange D, et al. Long-term effects of Internet-delivered cognitive behavioral therapy for depression in primary care - the PRIM-NET controlled trial. Scand J Prim Health Care 2017; 35: 126-136. |
| Wrong patient population | Farrer LM, Griffiths KM, Christensen H, Mackinnon AJ, Batterham PJ. Predictors of adherence and outcome in internet-based cognitive behavior therapy delivered in a telephone counseling setting. Cognitive Therapy and Research 2014; 38: 358-367. |
| Wrong patient population | Fitzpatrick KK, Darcy A, Vierhile M. Delivering Cognitive Behavior Therapy to Young Adults With Symptoms of Depression and Anxiety Using a Fully Automated Conversational Agent (Woebot): A Randomized Controlled Trial. JMIR Ment Health 2017; 4: e19. |
| Wrong patient population | Flett Jayde AM, Hayne Harlene, Riordan Benjamin C, Thompson Laura M, Conner Tamlin S. Mobile mindfulness meditation: A randomised controlled trial of the effect of two popular apps on mental health. Mindfulness 2018; : . |
| Wrong patient population | Fogarty AS, Proudfoot J, Whittle EL, Clarke J, Player MJ, Christensen H, Wilhelm K. Preliminary Evaluation of a Brief Web and Mobile Phone Intervention for Men With Depression: Men's Positive Coping Strategies and Associated Depression, Resilience, and Work and Social Functioning. JMIR Ment Health 2017; 4: e33. |
| Wrong patient population | Fogliati VJ, Dear BF, Staples LG, et al. Disorder-specific versus transdiagnostic and clinician-guided versus self-guided internet-delivered treatment for panic disorder and comorbid disorders: A randomized controlled trial. J Anxiety Disord 2016; 39: 88-102. |
| Wrong timeline | Fonzo GA, Fine NB, Wright RN, et al. Internet-delivered computerized cognitive & affective remediation training for the treatment of acute and chronic posttraumatic stress disorder: Two randomized clinical trials. J Psychiatr Res 2019; 115: 82-89. |
| Wrong patient population | Forand NR, Feinberg JE, Barnett JG, Strunk DR. Guided internet CBT versus "gold standard" depression treatments: An individual patient analysis. J Clin Psychol 2019; 75: 581-593. |
| Wrong patient population | Fortney JC, Pyne JM, Kimbrell TA, et al. Telemedicine-based collaborative care for posttraumatic stress disorder: a randomized clinical trial. JAMA Psychiatry 2015; 72: 58-67. |
| Wrong patient population | Franklin CL, Cuccurullo LA, Walton JL, Arseneau JR, Petersen NJ. Face to face but not in the same place: A pilot study of prolonged exposure therapy. J Trauma Dissociation 2017; 18: 116-130. |
| Wrong patient population | Furber G, Jones GM, Healey D, Bidargaddi N. A comparison between phone-based psychotherapy with and without text messaging support in between sessions for crisis patients. J Med Internet Res 2014; 16: e219. |
| Wrong outcomes | Furukawa TA, Horikoshi M, Fujita H, et al. Cognitive and Behavioral Skills Exercises Completed by Patients with Major Depression During Smartphone Cognitive Behavioral Therapy: Secondary Analysis of a Randomized Controlled Trial. JMIR Ment Health 2018; 5: e4. |
| Wrong patient population | G√∂rges F, Oehler C, von Hirschhausen E, Hegerl U, Rummel-Kluge C. GET.HAPPY - Acceptance of an internet-based self-management positive psychology intervention for adult primary care patients with mild and moderate depression or dysthymia: A pilot study. Internet Interventions 2018; 12: 26-35. |
| Wrong outcomes | Gallegos AM, Streltzov NA, Stecker T. Improving Treatment Engagement for Returning Operation Enduring Freedom and Operation Iraqi Freedom Veterans With Posttraumatic Stress Disorder, Depression, and Suicidal Ideation. J Nerv Ment Dis 2016; 204: 339-43. |
| Wrong patient population | Garnefski N, Kraaij V, Schroevers M. Effects of a cognitive behavioral self-help program on depressed mood for people with acquired chronic physical impairments: a pilot randomized controlled trial. Patient Educ Couns 2011; 85: 304-7. |
| Wrong intervention | Gawande R, Pine E, Griswold T, et al. Insurance-Reimbursable Mindfulness for Safety-Net Primary Care Patients: A Pilot Randomized Controlled Trial. Mindfulness (N Y) 2019; 10: 1744-1759. |
| Wrong patient population | Gellatly J, Chisnall L, Seccombe N, Ragan K, Lidbetter N, Cavanagh K. @Home eTherapy Service for People with Common Mental Health Problems: an Evaluation. Behav Cogn Psychother 2018; 46: 115-120. |
| Wrong patient population | Gellis ZD, Kenaley BL, Ten Have T. Integrated telehealth care for chronic illness and depression in geriatric home care patients: the Integrated Telehealth Education and Activation of Mood (I-TEAM) study. J Am Geriatr Soc 2014; 62: 889-95. |
| Wrong patient population | Geraedts AS, Kleiboer AM, Twisk J, Wiezer NM, van Mechelen W, Cuijpers P. Long-term results of a web-based guided self-help intervention for employees with depressive symptoms: randomized controlled trial. J Med Internet Res 2014; 16: e168. |
| Wrong indication | Geramita EM, Herbeck Belnap B, Abebe KZ, Rothenberger SD, Rotondi AJ, Rollman BL. The Association Between Increased Levels of Patient Engagement With an Internet Support Group and Improved Mental Health Outcomes at 6-Month Follow-Up: Post-Hoc Analyses From a Randomized Controlled Trial. J Med Internet Res 2018; 20: e10402. |
| Wrong patient population | Ghawadra SF, Lim Abdullah K, Choo WY, Danaee M, Phang CK. The effect of mindfulness-based training on stress, anxiety, depression and job satisfaction among ward nurses: A randomized control trial. J Nurs Manag 2020; 28: 1088-1097. |
| Wrong study design | Gilbody S, Brabyn S, Lovell K, et al. Telephone-supported computerised cognitive-behavioural therapy: REEACT-2 large-scale pragmatic randomised controlled trial. Br J Psychiatry 2017; 210: 362-367. |
| High Risk of Bias | Gili M, Castro A, Garc√≠a-Palacios A, et al. Efficacy of Three Low-Intensity, Internet-Based Psychological Interventions for the Treatment of Depression in Primary Care: Randomized Controlled Trial. J Med Internet Res 2020; 22: e15845. |
| Non English | Givi HG, Imani H, Agh A, Rik NM, Mehrabadi S. Efficiency of computerized cognitive behavioral therapy versus clinical intervention for the treatment of major depression. Koomesh 2012; 13: 218-224. |
| Wrong intervention | Glozier N, Christensen H, Griffiths KM, et al. Adjunctive Internet-delivered cognitive behavioural therapy for insomnia in men with depression: A randomised controlled trial. Aust N Z J Psychiatry 2019; 53: 350-360. |
| Wrong study design | Graham AK, Greene CJ, Kwasny MJ, Kaiser SM, Lieponis P, Powell T, Mohr DC. Coached Mobile App Platform for the Treatment of Depression and Anxiety Among Primary Care Patients: A Randomized Clinical Trial. JAMA Psychiatry 2020; 77: 906-914. |
| Wrong patient population | Griffiths KM, Mackinnon AJ, Crisp DA, Christensen H, Bennett K, Farrer L. The effectiveness of an online support group for members of the community with depression: a randomised controlled trial. PLoS One 2012; 7: e53244. |
| Wrong patient population | Gros DF, Yoder M, Tuerk PW, Lozano BE, Acierno R. Exposure Therapy for PTSD Delivered to Veterans via Telehealth: Predictors of Treatment Completion and Outcome and Comparison to Treatment Delivered in Person. Behavior Therapy 2011; 42: 276-283. |
| Wrong patient population | Hadjistavropoulos HD, Nugent MM, Alberts NM, Staples L, Dear BF, Titov N. Transdiagnostic Internet-delivered cognitive behaviour therapy in Canada: An open trial comparing results of a specialized online clinic and nonspecialized community clinics. J Anxiety Disord 2016; 42: 19-29. |
| Wrong patient population | Hadjistavropoulos HD, Schneider LH, Edmonds M, et al. Randomized controlled trial of internet-delivered cognitive behaviour therapy comparing standard weekly versus optional weekly therapist support. J Anxiety Disord 2017; 52: 15-24. |
| Wrong patient population | Hadjistavropoulos HD, Schneider LH, Edmonds M, et al. Randomized controlled trial of internet-delivered cognitive behaviour therapy comparing standard weekly versus optional weekly therapist support. J Anxiety Disord 2017; 52: 15-24. |
| Wrong patient population | Hadjistavropoulos HD, Schneider LH, Mehta S, Karin E, Dear BF, Titov N. Preference trial of internet-delivered cognitive behaviour therapy comparing standard weekly versus optional weekly therapist support. J Anxiety Disord 2019; 63: 51-60. |
| Wrong study design | Haller E, Besson N, Watzke B. "Unrigging the support wheels" - A qualitative study on patients' experiences with and perspectives on low-intensity CBT. BMC Health Serv Res 2019; 19: 686. |
| High Risk of Bias | Haller N, Lorenz S, Pfirrmann D, et al. Individualized Web-Based Exercise for the Treatment of Depression: Randomized Controlled Trial. JMIR Ment Health 2018; 5: e10698. |
| Duplicate Study | Haller N, Lorenz S, Pfirrmann D, et al. Individualized Web-Based Exercise for the Treatment of Depression: Randomized Controlled Trial. JMIR Ment Health 2018; 5: e10698. |
| Wrong patient population | Hallford DJ, Austin DW, Takano K, Fuller-Tyszkiewicz M, Raes F. Computerized Memory Specificity Training (c-MeST) for major depression: A randomised controlled trial. Behav Res Ther 2021; 136: 103783. |
| Wrong patient population | Hallgren M, Kraepelien M, Ojehagen A, Lindefors N, Zeebari Z, Kaldo V, Forsell Y. Physical exercise and internet-based cognitive-behavioural therapy in the treatment of depression: randomised controlled trial. Br J Psychiatry 2015; 207: 227-34. |
| Wrong outcomes | Hatcher S, Whittaker R, Patton M, Miles WS, Ralph N, Kercher K, Sharon C. Web-based Therapy Plus Support by a Coach in Depressed Patients Referred to Secondary Mental Health Care: Randomized Controlled Trial. JMIR Ment Health 2018; 5: e5. |
| Wrong patient population | Hedman E, Andersson G, Andersson E, Ljotsson B, Ruck C, Asmundson GJ, Lindefors N. Internet-based cognitive-behavioural therapy for severe health anxiety: randomised controlled trial. Br J Psychiatry 2011; 198: 230-6. |
| Wrong study design | Hedman E, Ljotsson B, Kaldo V, et al. Effectiveness of Internet-based cognitive behaviour therapy for depression in routine psychiatric care. J Affect Disord 2014; 155: 49-58. |
| Wrong type of publication | Hegerl U, Oehler C. Promises and risks of web-based interventions in the treatment of depression‚Ä©. Dialogues Clin Neurosci 2020; 22: 161-168. |
| Wrong study design | Herrera-Mercadal P, Montero-Marin J, Plaza I, et al. The efficacy and pattern of use of a computer-assisted programme for the treatment of anxiety: a naturalistic study using mixed methods in primary care in Spain. J Affect Disord 2015; 175: 184-91. |
| Wrong patient population | Hetrick SE, Yuen HP, Bailey E, et al. Internet-based cognitive behavioural therapy for young people with suicide-related behaviour (Reframe-IT): a randomised controlled trial. Evid Based Ment Health 2017; 20: 76-82. |
| Wrong study design | Himelhoch S, Mohr D, Maxfield J, Clayton S, Weber E, Medoff D, Dixon L. Feasibility of telephone-based cognitive behavioral therapy targeting major depression among urban dwelling African-American people with co-occurring HIV. Psychol Health Med 2011; 16: 156-65. |
| Wrong patient population | Hinkle JF. The Stress Gym: An Online Intervention to Improve Stress and Depressive Symptoms in Adults. Issues Ment Health Nurs 2015; 36: 870-6. |
| Wrong patient population | Hirani SP, Beynon M, Cartwright M, et al. The effect of telecare on the quality of life and psychological well-being of elderly recipients of social care over a 12-month period: the Whole Systems Demonstrator cluster randomised trial. Age Ageing 2014; 43: 334-41. |
| Wrong patient population | Hiratsuka VY, Moore L, Avey JP, Dirks LG, Beach BD, Dillard DA, Novins DK. An Internet-Based Therapeutic Tool for American Indian/Alaska Native Adults With Posttraumatic Stress Disorder: User Testing and Developmental Feasibility Study. JMIR Form Res 2019; 3: e13682. |
| Wrong patient population | Hirsch A, Luellen J, Holder JM, Steinberg G, Dubiel T, Blazejowskyj A, Schladweiler K. Managing Depressive Symptoms in the Workplace Using a Web-Based Self-Care Tool: A Pilot Randomized Controlled Trial. JMIR Res Protoc 2017; 6: e51. |
| High Risk of Bias | Hirsch CR, Krahe C, Whyte J, Loizou S, Bridge L, Norton S, Mathews A. Interpretation training to target repetitive negative thinking in generalized anxiety disorder and depression. J Consult Clin Psychol 2018; 86: 1017-1030. |
| Wrong patient population | Hobfoll SE, Blais RK, Stevens NR, Walt L, Gengler R. Vets prevail online intervention reduces PTSD and depression in veterans with mild-to-moderate symptoms. J Consult Clin Psychol 2016; 84: 31-42. |
| Wrong patient population | Hoerster KD, Jakupcak M, Stephenson KR, et al. A pilot trial of telephone-based collaborative care management for PTSD among Iraq/Afghanistan war veterans. Telemed J E Health 2015; 21: 42-7. |
| Wrong patient population | Hoffmann D, Rask CU, Hedman-Lagerlof E, Ljotsson B, Frostholm L. Development and Feasibility Testing of Internet-Delivered Acceptance and Commitment Therapy for Severe Health Anxiety: Pilot Study. JMIR Ment Health 2018; 5: e28. |
| High Risk of Bias | Hoifodt RS, Lillevoll KR, Griffiths KM, Wilsgaard T, Eisemann M, Waterloo K, Kolstrup N. The clinical effectiveness of web-based cognitive behavioral therapy with face-to-face therapist support for depressed primary care patients: randomized controlled trial. J Med Internet Res 2013; 15: e153. |
| Wrong patient population | Holl√§ndare F, Johnsson S, Randestad M, Tillfors M, Carlbring P, Andersson G, Engstr√∂m I. Randomized trial of internet‚Äêbased relapse prevention for partially remitted depression. Acta Psychiatrica Scandinavica 2011; 124: 285-294. |
| Wrong patient population | Holl√§ndare Fredrik, Anthony Susanne A, Randestad Mia, Tillfors Maria, Carlbring Per, Andersson Gerhard, Engstr√∂m Ingemar. Two-year outcome of internet-based relapse prevention for partially remitted depression. Behaviour Research and Therapy 2013; 51: 719-722. |
| Wrong patient population | Huberty J, Sullivan M, Green J, Kurka J, Leiferman J, Gold K, Cacciatore J. Online yoga to reduce post traumatic stress in women who have experienced stillbirth: a randomized control feasibility trial. BMC Complement Med Ther 2020; 20: 173. |
| Wrong study design | Hull TD, Malgaroli M, Connolly PS, Feuerstein S, Simon NM. Two-way messaging therapy for depression and anxiety: longitudinal response trajectories. BMC Psychiatry 2020; 20: 297. |
| Wrong study design | Hunkeler EM, Hargreaves WA, Fireman B, et al. A web-delivered care management and patient self-management program for recurrent depression: a randomized trial. Psychiatr Serv 2012; 63: 1063-71. |
| Wrong study design | Hur JW, Kim B, Park D, Choi SW. A Scenario-Based Cognitive Behavioral Therapy Mobile App to Reduce Dysfunctional Beliefs in Individuals with Depression: A Randomized Controlled Trial. Telemed J E Health 2018; 24: 710-716. |
| Wrong patient population | Hwang WJ, Jo HH. Evaluation of the Effectiveness of Mobile App-Based Stress-Management Program: A Randomized Controlled Trial. Int J Environ Res Public Health 2019; 16: . |
| Wrong intervention | Iacoviello BM, Murrough JW, Hoch MM, et al. A randomized, controlled pilot trial of the Emotional Faces Memory Task: a digital therapeutic for depression. NPJ Digit Med 2018; 1: . |
| Wrong type of publication | Imamura K, Kawakami N, Furukawa TA, Matsuyama Y, Shimazu A, Kasai K. Effects of an internet-based cognitive behavioural therapy intervention on preventing major depressive episodes among workers: A protocol for a randomised controlled trial. BMJ Open 2015; 5: . |
| Wrong patient population | Imamura K, Kawakami N, Tsuno K, Tsuchiya M, Shimada K, Namba K. Effects of web-based stress and depression literacy intervention on improving symptoms and knowledge of depression among workers: A randomized controlled trial. J Affect Disord 2016; 203: 30-37. |
| Wrong setting | Imamura K, Tran TTT, Nguyen HT, et al. Effects of two types of smartphone-based stress management programmes on depressive and anxiety symptoms among hospital nurses in Vietnam: A protocol for three-arm randomised controlled trial. BMJ Open 2019; 9: . |
| Wrong patient population | Ivarsson D, Blom M, Hesser H, Carlbring P, Enderby P, Nordberg R, Andersson G. Guided internet-delivered cognitive behavior therapy for post-traumatic stress disorder: A randomized controlled trial. Internet Interventions 2014; 1: 33-40. |
| Wrong patient population | Jahoda A, Hastings R, Hatton C, et al. Behavioural activation versus guided self-help for depression in adults with learning disabilities: the BeatIt RCT. Health Technol Assess 2018; 22: 1-130. |
| Wrong setting | Jannati N, Mazhari S, Ahmadian L, Mirzaee M. Effectiveness of an app-based cognitive behavioral therapy program for postpartum depression in primary care: A randomized controlled trial. Int J Med Inform 2020; 141: 104145. |
| Wrong patient population | Jelinek L, Arlt S, Moritz S, Schroder J, Westermann S, Cludius B. Brief Web-Based Intervention for Depression: Randomized Controlled Trial on Behavioral Activation. J Med Internet Res 2020; 22: e15312. |
| Wrong patient population | Jensen CG, Lansner J, Petersen A, et al. Open and Calm--a randomized controlled trial evaluating a public stress reduction program in Denmark. BMC Public Health 2015; 15: 1245. |
| Wrong patient population | Johansson P, Jaarsma T, Andersson G, Lundgren J. The impact of internet-based cognitive behavioral therapy and depressive symptoms on self-care behavior in patients with heart failure. A secondary analysis of a randomised controlled trial. Int J Nurs Stud 2019; : 103454. |
| Wrong patient population | Johansson R, Bjorklund M, Hornborg C, et al. Affect-focused psychodynamic psychotherapy for depression and anxiety through the Internet: a randomized controlled trial. PeerJ 2013; 1: e102. |
| Wrong outcomes | Johansson R, Hesser H, Lj√≥tsson B, Frederick RJ, Andersson G. Transdiagnostic, affect-focused, psychodynamic, guided self-help for depression and anxiety through the internet: Study protocol for a randomised controlled trial. BMJ Open 2012; 2: . |
| Wrong patient population | Johansson R, Nyblom A, Carlbring P, Cuijpers P, Andersson G. Choosing between Internet-based psychodynamic versus cognitive behavioral therapy for depression: a pilot preference study. BMC Psychiatry 2013; 13: 268. |
| Wrong patient population | Johansson R, Sjoberg E, Sjogren M, et al. Tailored vs. standardized internet-based cognitive behavior therapy for depression and comorbid symptoms: a randomized controlled trial. PLoS One 2012; 7: e36905. |
| Wrong patient population | Johnston L, Titov N, Andrews G, Spence J, Dear BF. A RCT of a transdiagnostic internet-delivered treatment for three anxiety disorders: examination of support roles and disorder-specific outcomes. PLoS One 2011; 6: e28079. |
| Wrong study design | Jonassaint CR, Belnap BH, Huang Y, Karp JF, Abebe KZ, Rollman BL. Racial Differences in the Effectiveness of Internet-Delivered Mental Health Care. J Gen Intern Med 2020; 35: 490-497. |
| Wrong patient population | Jones SL, Hadjistavropoulos HD, Soucy JN. A randomized controlled trial of guided internet-delivered cognitive behaviour therapy for older adults with generalized anxiety. J Anxiety Disord 2016; 37: 44570. |
| Study Design Only - No Results | Justicia A, Elices M, Cebria AI, et al. Rationale and methods of the iFightDepression study: A double-blind, randomized controlled trial evaluating the efficacy of an internet-based self-management tool for moderate to mild depression. BMC Psychiatry 2017; 17: 143. |
| Wrong patient population | K√§hlke Fanny, Berger Thomas, Schulz Ava, et al. Efficacy of an unguided internet‚Äêbased self‚Äêhelp intervention for social anxiety disorder in university students: A randomized controlled trial. International Journal of Methods in Psychiatric Research 2019; : . |
| Wrong patient population | Kageyama K, Kato Y, Mesaki T, et al. Effects of video viewing smartphone application intervention involving positive word stimulation in people with subthreshold depression: A pilot randomized controlled trial. J Affect Disord 2021; 282: 74-81. |
| Wrong patient population | Kahn JR, Collinge W, Soltysik R. Post-9/11 Veterans and Their Partners Improve Mental Health Outcomes with a Self-directed Mobile and Web-based Wellness Training Program: A Randomized Controlled Trial. J Med Internet Res 2016; 18: e255. |
| Wrong outcomes | Kaldo V, Lundin A, Hallgren M, et al. Effects of internet-based cognitive behavioural therapy and physical exercise on sick leave and employment in primary care patients with depression: two subgroup analyses. Occup Environ Med 2018; 75: 52-58. |
| Study Design Only - No Results | Kanuri N, Newman MG, Ruzek JI, et al. The Feasibility, Acceptability, and Efficacy of Delivering Internet-Based Self-Help and Guided Self-Help Interventions for Generalized Anxiety Disorder to Indian University Students: Design of a Randomized Controlled Trial. JMIR Res Protoc 2015; 4: e136. |
| Wrong patient population | Kelders SM, Bohlmeijer ET, Pots WT, van Gemert-Pijnen JE. Comparing human and automated support for depression: Fractional factorial randomized controlled trial. Behav Res Ther 2015; 72: 72-80. |
| Study Design Only - No Results | Kenter RMF, van Straten A, Hobbel SH, Smit F, Bosmans J, Beekman A, Cuijpers P. Effectiveness and cost effectiveness of guided online treatment for patients with major depressive disorder on a waiting list for psychotherapy: Study protocol of a randomized controlled trial. Trials 2013; 14: . |
| Wrong patient population | Kersting A, Dolemeyer R, Steinig J, Walter F, Kroker K, Baust K, Wagner B. Brief Internet-based intervention reduces posttraumatic stress and prolonged grief in parents after the loss of a child during pregnancy: a randomized controlled trial. Psychother Psychosom 2013; 82: 372-81. |
| Wrong patient population | Kersting A, Kroker K, Schlicht S, Baust K, Wagner B. Efficacy of cognitive behavioral internet-based therapy in parents after the loss of a child during pregnancy: Pilot data from a randomized controlled trial. Archives of Women's Mental Health 2011; 14: 465-477. |
| Wrong patient population | Khatri N, Marziali E, Tchernikov I, Shepherd N. Comparing telehealth-based and clinic-based group cognitive behavioral therapy for adults with depression and anxiety: a pilot study. Clin Interv Aging 2014; 9: 765-70. |
| Wrong patient population | Khazaeili Mahnaz, Hajebi Majid Zargham, Mohamadkhani Parvaneh, Mirzahoseini Hasan. The effectiveness of mindfulness-based Internet intervention on the anxiety, depression, and fatigue of the patients with multiple sclerosis. Journal of Practice in Clinical Psychology 2019; 7: 137-146. |
| Wrong study design | Kiosses DN, Alexopoulos GS. Problem-Solving Therapy in the Elderly. Curr Treat Options Psychiatry 2014; 1: 15-26. |
| Wrong intervention | Kivelitz L, Kriston L, Christalle E, et al. Effectiveness of telephone-based aftercare case management for adult patients with unipolar depression compared to usual care: A randomized controlled trial. PLoS One 2017; 12: e0186967. |
| Wrong patient population | Kivi M, Eriksson MC, Hange D, Petersson EL, Vernmark K, Johansson B, Bjorkelund C. Internet-based therapy for mild to moderate depression in Swedish primary care: short term results from the PRIM-NET randomized controlled trial. Cogn Behav Ther 2014; 43: 289-98. |
| Wrong patient population | Kladnitski N, Smith J, Uppal S, James MA, Allen AR, Andrews G, Newby JM. Transdiagnostic internet-delivered CBT and mindfulness-based treatment for depression and anxiety: A randomised controlled trial. Internet Interv 2020; 20: 100310. |
| Wrong patient population | Kleiboer A, Donker T, Seekles W, van Straten A, Riper H, Cuijpers P. A randomized controlled trial on the role of support in Internet-based problem solving therapy for depression and anxiety. Behav Res Ther 2015; 72: 63-71. |
| Wrong patient population | Klein B, Meyer D, Austin DW, Kyrios M. Anxiety online: a virtual clinic: preliminary outcomes following completion of five fully automated treatment programs for anxiety disorders and symptoms. J Med Internet Res 2011; 13: e89. |
| Wrong study design | Klein B, Mitchell J, Abbott J, et al. A therapist-assisted cognitive behavior therapy internet intervention for posttraumatic stress disorder: pre-, post- and 3-month follow-up results from an open trial. J Anxiety Disord 2010; 24: 635-44. |
| Wrong patient population | Klein JP, Spath C, Schroder J, et al. Time to remission from mild to moderate depressive symptoms: One year results from the EVIDENT-study, an RCT of an internet intervention for depression. Behav Res Ther 2017; 97: 154-162. |
| Wrong patient population | Knaevelsrud C, B√∂ttche M, Pietrzak RH, Freyberger HJ, Renneberg B, Kuwert P. An internet-based, therapist-assisted therapy for german elderly survivors of the world war II with posttraumatic stress symptoms. Journal of Nervous and Mental Disease 2014; 202: 651-658. |
| Wrong patient population | Knaevelsrud C, Bottche M, Pietrzak RH, Freyberger HJ, Renneberg B, Kuwert P. Integrative testimonial therapy: an Internet-based, therapist-assisted therapy for German elderly survivors of the World War II with posttraumatic stress symptoms. J Nerv Ment Dis 2014; 202: 651-8. |
| Wrong patient population | Knaevelsrud C, Brand J, Lange A, Ruwaard J, Wagner B. Web-based psychotherapy for posttraumatic stress disorder in war-traumatized Arab patients: randomized controlled trial. J Med Internet Res 2015; 17: e71. |
| Wrong patient population | Knaevelsrud C, Maercker A. Long-term effects of an internet-based treatment for posttraumatic stress. Cogn Behav Ther 2010; 39: 72-7. |
| Wrong patient population | Kok G, Burger H, Riper H, et al. The Three-Month Effect of Mobile Internet-Based Cognitive Therapy on the Course of Depressive Symptoms in Remitted Recurrently Depressed Patients: Results of a Randomized Controlled Trial. Psychother Psychosom 2015; 84: 90-99. |
| Study Design Only - No Results | Kordy H, Backenstrass M, Husing J, et al. Supportive monitoring and disease management through the internet: an internet-delivered intervention strategy for recurrent depression. Contemp Clin Trials 2013; 36: 327-37. |
| Wrong patient population | Kordy H, Wolf M, Aulich K, et al. Internet-Delivered Disease Management for Recurrent Depression: A Multicenter Randomized Controlled Trial. Psychother Psychosom 2016; 85: 91-8. |
| Wrong patient population | Kr√§mer LV, Gr√ºnzig SD, Baumeister H, Ebert DD, Bengel J. Effectiveness of a Guided Web-Based Intervention to Reduce Depressive Symptoms before Outpatient Psychotherapy: A Pragmatic Randomized Controlled Trial. Psychother Psychosom 2021; 90: 233-242. |
| Wrong indication | Kratz AL, Ehde DM, Bombardier CH. Affective mediators of a physical activity intervention for depression in multiple sclerosis. Rehabil Psychol 2014; 59: 57-67. |
| Wrong intervention | Krejtz I, Holas P, Rusanowska M, Nezlek JB. Positive online attentional training as a means of modifying attentional and interpretational biases among the clinically depressed: An experimental study using eye tracking. J Clin Psychol 2018; 74: 1594-1606. |
| Wrong intervention | Krieger T, Meyer B, Sude K, Urech A, Maercker A, Berger T. Evaluating an e-mental health program ("deprexis") as adjunctive treatment tool in psychotherapy for depression: design of a pragmatic randomized controlled trial. BMC Psychiatry 2014; 14: 285. |
| Wrong patient population | Krupnick JL, Green BL, Amdur R, et al. An Internet-based writing intervention for PTSD in veterans: A feasibility and pilot effectiveness trial. Psychol Trauma 2017; 9: 461-470. |
| Wrong indication | Kuckertz JM, Gildebrant E, Liliequist B, et al. Moderation and mediation of the effect of attention training in social anxiety disorder. Behav Res Ther 2014; 53: 30-40. |
| Wrong study design | Kumar S, Tran JLA, Ramirez E, Lee WN, Foschini L, Juusola JL. Design, Recruitment, and Baseline Characteristics of a Virtual 1-Year Mental Health Study on Behavioral Data and Health Outcomes: Observational Study. JMIR Ment Health 2020; 7: e17075. |
| Non English | L√∂bner Margrit, Dorow Marie, Pabst Alexander, et al. Online-Selbstmanagement bei Depressionen‚ÄîDie Beziehung von Dosis und Wirksamkeit im haus√§rztlichen Versorgungssetting = Online self-management of depression‚ÄîDose-response relationship within a primary care setting. Fortschritte der Neurologie, Psychiatrie 2019; 87: 181-186. |
| Wrong patient population | L√≥pez Georgina C√°rdenas, Arbona Cristina Botella, Castellano Soledad Quero, G√≥mez Anabel de la Rosa, Rivera Rosa Mar√≠a Ba√±os. Programa de telepsicolog√≠a para el tratamiento de la fobia a hablar en p√∫blico en poblaci√≥n Mexicana = Telepsychology program to treat public-speaking anxiety among the Mexican population. Psicolog√≠a Iberoamericana 2014; 22: 45-54. |
| Wrong patient population | Lam RW, Parikh SV, Ramasubbu R, et al. Effects of combined pharmacotherapy and psychotherapy for improving work functioning in major depressive disorder. Br J Psychiatry 2013; 203: 358-65. |
| Wrong patient population | Lappalainen P, Granlund A, Siltanen S, Ahonen S, Vitikainen M, Tolvanen A, Lappalainen R. ACT Internet-based vs face-to-face? A randomized controlled trial of two ways to deliver Acceptance and Commitment Therapy for depressive symptoms: an 18-month follow-up. Behav Res Ther 2014; 61: 43-54. |
| Wrong patient population | Lappalainen P, Granlund A, Siltanen S, Ahonen S, Vitikainen M, Tolvanen A, Lappalainen R. ACT Internet-based vs face-to-face? A randomized controlled trial of two ways to deliver Acceptance and Commitment Therapy for depressive symptoms: an 18-month follow-up. Behav Res Ther 2014; 61: 43-54. |
| Wrong patient population | Lappalainen P, Langrial S, Oinas-Kukkonen H, Tolvanen A, Lappalainen R. Web-based acceptance and commitment therapy for depressive symptoms with minimal support: a randomized controlled trial. Behav Modif 2015; 39: 805-34. |
| Paediatric population | Lappalainen R, Lappalainen P, Puolakanaho A, et al. The Youth Compass - the effectiveness of an online acceptance and commitment therapy program to promote adolescent mental health: A randomized controlled trial. Journal of Contextual Behavioral Science 2021; 20: 44573. |
| Wrong patient population | Lauder S, Chester A, Castle D, et al. A randomized head to head trial of MoodSwings.net.au: an Internet based self-help program for bipolar disorder. J Affect Disord 2015; 171: 13-21. |
| Wrong study design | Lee WJ, Choi SH, Shin JE, et al. Effects of an online imagery-based treatment program in patients with workplace-related posttraumatic stress disorder: A pilot study. Psychiatry Investigation 2018; 15: 1071-1078. |
| Wrong patient population | Lemma A, Fonagy P. Feasibility study of a psychodynamic online group intervention for depression. Psychoanalytic Psychology 2013; 30: 367-380. |
| Wrong study design | Lerner D, Adler D, Shayani A, Rogers WH. Research on the Tufts Be Well at Work Program for Employees With Depression: 2005-2020. Psychiatr Serv 2021; : appips202000762. |
| Wrong patient population | Lewis Catrin E, Farewell Daniel, Groves Vicky, Kitchiner Neil J, Roberts Neil P, Vick Tracey, Bisson Jonathan I. Internet‚Äêbased guided self‚Äêhelp for posttraumatic stress disorder (PTSD): Randomized controlled trial. Depression and Anxiety 2017; 34: 555-565. |
| Wrong patient population | Li Y, Guo Y, Hong YA, et al. Mechanisms and Effects of a WeChat-Based Intervention on Suicide Among People Living With HIV and Depression: Path Model Analysis of a Randomized Controlled Trial. J Med Internet Res 2019; 21: e14729. |
| Wrong indication | Lindegaard T, Seaton F, Halaj A, et al. Internet-based cognitive behavioural therapy for depression and anxiety among Arabic-speaking individuals in Sweden: a pilot randomized controlled trial. Cogn Behav Ther 2020; : 44581. |
| Wrong study design | Lindner P, Olsson EL, Johnsson A, Dahlin M, Andersson G, Carlbring P. The impact of telephone versus e-mail therapist guidance on treatment outcomes, therapeutic alliance and treatment engagement in Internet-delivered CBT for depression: A randomised pilot trial. Internet Interventions 2014; 1: 182-187. |
| Wrong patient population | Littlewood E, Duarte A, Hewitt C, et al. A randomised controlled trial of computerised cognitive behaviour therapy for the treatment of depression in primary care: the Randomised Evaluation of the Effectiveness and Acceptability of Computerised Therapy (REEACT) trial. Health Technol Assess 2015; 19: viii, xxi-171. |
| Wrong study design | Lokman S, Leone SS, Sommers-Spijkerman M, van der Poel A, Smit F, Boon B. Complaint-Directed Mini-Interventions for Depressive Complaints: A Randomized Controlled Trial of Unguided Web-Based Self-Help Interventions. J Med Internet Res 2017; 19: e4. |
| Study Design Only - No Results | Lopez-del-Hoyo Y, Olivan B, Luciano JV, et al. Low intensity vs. self-guided internet-delivered psychotherapy for major depression: a multicenter, controlled, randomized study. BMC Psychiatry 2013; 13: 21. |
| Study Design Only - No Results | Lopez-Montoyo A, Quero S, Montero-Marin J, Barcelo-Soler A, Beltran M, Campos D, Garcia-Campayo J. Effectiveness of a brief psychological mindfulness-based intervention for the treatment of depression in primary care: Study protocol for a randomized controlled clinical trial. BMC Psychiatry 2019; 19: . |
| Wrong patient population | Loughnan Siobhan A, Butler Christine, Sie Amanda A, et al. A randomised controlled trial of ‚ÄòMUMentum postnatal‚Äô: Internet-delivered cognitive behavioural therapy for anxiety and depression in postpartum women. Behaviour Research and Therapy 2019; 116: 94-103. |
| Wrong patient population | Lu SHX, Assudani HA, Kwek TRR, et al., Teoh TEL, Tan GCY. A Randomised Controlled Trial of Clinician-Guided Internet-Based Cognitive Behavioural Therapy for Depressed Patients in Singapore. Front Psychol 2021; 12: 668384. |
| Wrong outcomes | Ludtke T, Westermann S, Pult LK, Schneider BC, Pfuhl G, Moritz S. Evaluation of a brief unguided psychological online intervention for depression: A controlled trial including exploratory moderator analyses. Internet Interv 2018; 13: 73-81. |
| Wrong patient population | Lungu A, Jun JJ, Azarmanesh O, Leykin Y, Chen CE. Blended Care-Cognitive Behavioral Therapy for Depression and Anxiety in Real-World Settings: Pragmatic Retrospective Study. J Med Internet Res 2020; 22: e18723. |
| Wrong patient population | Luxton DD, Pruitt LD, Wagner A, Smolenski DJ, Jenkins-Guarnieri MA, Gahm G. Home-based telebehavioral health for U.S. military personnel and veterans with depression: A randomized controlled trial. J Consult Clin Psychol 2016; 84: 923-934. |
| Wrong intervention | Ly KH, Topooco N, Cederlund H, et al. Smartphone-Supported versus Full Behavioural Activation for Depression: A Randomised Controlled Trial. PLoS One 2015; 10: e0126559. |
| Paediatric population | MacLean S, Corsi DJ, Litchfield S, et al. Coach-Facilitated Web-Based Therapy Compared With Information About Web-Based Resources in Patients Referred to Secondary Mental Health Care for Depression: Randomized Controlled Trial. J Med Internet Res 2020; 22: e15001. |
| Wrong patient population | Madsen T, van Spijker B, Karstoft KI, Nordentoft M, Kerkhof AJ. Trajectories of Suicidal Ideation in People Seeking Web-Based Help for Suicidality: Secondary Analysis of a Dutch Randomized Controlled Trial. J Med Internet Res 2016; 18: e178. |
| Wrong patient population | Maieritsch KP, Smith TL, Hessinger JD, Ahearn EP, Eickhoff JC, Zhao Q. Randomized controlled equivalence trial comparing videoconference and in person delivery of cognitive processing therapy for PTSD. J Telemed Telecare 2016; 22: 238-43. |
| Wrong patient population | Mailey EL, Wojcicki TR, Motl RW, Hu L, Strauser DR, Collins KD, McAuley E. Internet-delivered physical activity intervention for college students with mental health disorders: a randomized pilot trial. Psychol Health Med 2010; 15: 646-59. |
| Wrong study design | Mantani A, Kato T, Furukawa TA, et al. Smartphone Cognitive Behavioral Therapy as an Adjunct to Pharmacotherapy for Refractory Depression: Randomized Controlled Trial. J Med Internet Res 2017; 19: e373. |
| Duplicate Study | Mantani Akio, Kato Tadashi, Furukawa Toshi A, et al. Correction: 'Smartphone cognitive behavioral therapy as an adjunct to pharmacotherapy for refractory depression: Randomized controlled trial'. Journal of Medical Internet Research 2018; 20: . |
| Wrong setting | Marasinghe RB, Edirippulige S, Kavanagh D, Smith A, Jiffry MT. Effect of mobile phone-based psychotherapy in suicide prevention: a randomized controlled trial in Sri Lanka. J Telemed Telecare 2012; 18: 151-5. |
| Wrong type of publication | Maresova P, Klimova B, Kuca K. Mobile applications as good intervention tools for individuals with depression. Ceska Slov Farm 2017; 66: 55-61. |
| Wrong study design | Mathiasen K, Riper H, Andersen TE, Roessler KK. Guided Internet-Based Cognitive Behavioral Therapy for Adult Depression and Anxiety in Routine Secondary Care: Observational Study. J Med Internet Res 2018; 20: e10927. |
| Wrong study design | Maust DT, Mavandadi S, Benson A, et al. Telephone-based care management for older adults initiated on psychotropic medication. Int J Geriatr Psychiatry 2013; 28: 410-6. |
| Wrong patient population | Mavandadi S, Benson A, DiFilippo S, Streim JE, Oslin D. A Telephone-Based Program to Provide Symptom Monitoring Alone vs Symptom Monitoring Plus Care Management for Late-Life Depression and Anxiety: A Randomized Clinical Trial. JAMA Psychiatry 2015; 72: 1211-8. |
| Wrong outcomes | Meglic M, Furlan M, Kuzmanic M, et al. Feasibility of an eHealth service to support collaborative depression care: results of a pilot study. J Med Internet Res 2010; 12: e63. |
| Wrong patient population | Melnyk Bernadette Mazurek, Amaya Megan, Szalacha Laura A, Hoying Jacqueline, Taylor Tiffany, Bowersox Kristen. Feasibility, acceptability, and preliminary effects of the COPE online cognitive‚Äêbehavioral skill‚Äêbuilding program on mental health outcomes and academic performance in freshmen college students: A randomized controlled pilot study. Journal of Child and Adolescent Psychiatric Nursing 2015; 28: 147-154. |
| Study Design Only - No Results | Messerli-B√ºrgy N, Barth J, Berger T. The InterHerz project - a web-based psychological treatment for cardiac patients with depression: Study protocol of a randomized controlled trial. Trials 2012; 13: . |
| Wrong study design | Mewton L, Andrews G. Cognitive behaviour therapy via the internet for depression: A useful strategy to reduce suicidal ideation. Journal of Affective Disorders 2015; 170: 78-84. |
| Wrong patient population | Meyer B, Bierbrodt J, Schr√∂der J, et al. Effects of an Internet intervention (Deprexis) on severe depression symptoms: Randomized controlled trial. Internet Interventions 2015; 2: 48-59. |
| Wrong patient population | Meyer B, Weiss M, Holtkamp M, et al. Effects of an epilepsy-specific Internet intervention (Emyna) on depression: Results of the ENCODE randomized controlled trial. Epilepsia 2019; 60: 656-668. |
| Non English | Miegel Franziska, Gehlenborg Josefine, B√ºcker Lara, Lion Despina, Moritz Steffen. Kann eine online-intervention f√ºr depressionen emotionale probleme und schmerzen lindern? Eine randomisiert-kontrollierte studie = Can an online intervention for depression alleviate emotional problems and pain? A randomized controlled study. Verhaltenstherapie 2019; 29: 166-181. |
| Wrong patient population | Miner A, Kuhn E, Hoffman JE, Owen JE, Ruzek JI, Taylor CB. Feasibility, acceptability, and potential efficacy of the PTSD Coach app: A pilot randomized controlled trial with community trauma survivors. Psychol Trauma 2016; 8: 384-392. |
| Wrong patient population | Mira A, Breton-Lopez J, Garcia-Palacios A, Quero S, Banos RM, Botella C. An Internet-based program for depressive symptoms using human and automated support: a randomized controlled trial. Neuropsychiatr Dis Treat 2017; 13: 987-1006. |
| Wrong outcomes | Mira A, Soler C, Alda M, et al. Exploring the Relationship Between the Acceptability of an Internet-Based Intervention for Depression in Primary Care and Clinical Outcomes: Secondary Analysis of a Randomized Controlled Trial. Front Psychiatry 2019; 10: 325. |
| Wrong patient population | Moberg C, Niles A, Beermann D. Guided Self-Help Works: Randomized Waitlist Controlled Trial of Pacifica, a Mobile App Integrating Cognitive Behavioral Therapy and Mindfulness for Stress, Anxiety, and Depression. J Med Internet Res 2019; 21: e12556. |
| Wrong indication | Mohr DC, Carmody T, Erickson L, Jin L, Leader J. Telephone-administered cognitive behavioral therapy for veterans served by community-based outpatient clinics. J Consult Clin Psychol 2011; 79: 261-5. |
| High Risk of Bias | Montero-Marin J, Araya R, Perez-Yus MC, et al. An Internet-Based Intervention for Depression in Primary Care in Spain: A Randomized Controlled Trial. J Med Internet Res 2016; 18: e231. |
| Wrong patient population | Moritz S, Schilling L, Hauschildt M, Schroder J, Treszl A. A randomized controlled trial of internet-based therapy in depression. Behav Res Ther 2012; 50: 513-21. |
| Wrong intervention | Morland LA, Greene CJ, Rosen CS, et al. Telemedicine for anger management therapy in a rural population of combat veterans with posttraumatic stress disorder: a randomized noninferiority trial. J Clin Psychiatry 2010; 71: 855-63. |
| Wrong patient population | Morland LA, Hynes AK, Mackintosh MA, Resick PA, Chard KM. Group cognitive processing therapy delivered to veterans via telehealth: a pilot cohort. J Trauma Stress 2011; 24: 465-9. |
| Wrong patient population | Morland LA, Mackintosh MA, Glassman LH, et al. Home-based delivery of variable length prolonged exposure therapy: A comparison of clinical efficacy between service modalities. Depress Anxiety 2020; 37: 346-355. |
| Wrong patient population | Morland LA, Mackintosh MA, Greene CJ, Rosen CS, Chard KM, Resick P, Frueh BC. Cognitive processing therapy for posttraumatic stress disorder delivered to rural veterans via telemental health: a randomized noninferiority clinical trial. J Clin Psychiatry 2014; 75: 470-6. |
| Wrong patient population | Morland Leslie A, Mackintosh Margaret‚ÄêAnne, Glassman Lisa H, et al. Home‚Äêbased delivery of variable length prolonged exposure therapy: A comparison of clinical efficacy between service modalities. Depression and Anxiety 2019; : . |
| Wrong patient population | Morland Leslie A, Mackintosh Margaret‚ÄêAnne, Rosen Craig S, Willis Emy, Resick Patricia, Chard Kathleen, Frueh BChristopher. Telemedicine versus in‚Äêperson delivery of cognitive processing therapy for women with posttraumatic stress disorder: A randomized noninferiority trial. Depression and Anxiety 2015; 32: 811-820. |
| Wrong patient population | Morris J, Firkins A, Millings A, Mohr C, Redford P, Rowe A. Internet-delivered cognitive behavior therapy for anxiety and insomnia in a higher education context. Anxiety Stress Coping 2016; 29: 415-31. |
| Wrong patient population | Morriss R, Kaylor-Hughes C, Rawsthorne M, et al. A Direct-to-Public Peer Support Program (Big White Wall) Versus Web-Based Information to Aid the Self-management of Depression and Anxiety: Results and Challenges of an Automated Randomized Controlled Trial. J Med Internet Res 2021; 23: e23487. |
| Wrong patient population | Morriss R, Patel S, Malins S, et al. Clinical and economic outcomes of remotely delivered cognitive behaviour therapy versus treatment as usual for repeat unscheduled care users with severe health anxiety: a multicentre randomised controlled trial. BMC Med 2019; 17: 16. |
| Wrong type of publication | Morthland Martin, Shah Avani, Meadows James T, Scogin Forrest. Development of an audio and computer cognitive behavioral therapy for depression in older adults. Aging & Mental Health 2020; 24: 1207-1215. |
| Wrong intervention | Moskowitz JT, Addington EL, Shiu E, et al.. Facilitator Contact, Discussion Boards, and Virtual Badges as Adherence Enhancements to a Web-Based, Self-guided, Positive Psychological Intervention for Depression: Randomized Controlled Trial. J Med Internet Res 2021; 23: e25922. |
| Wrong patient population | Mota Pereira Jorge, Fonte Daniela, Teixeira Ricardo Jo√£o, Carvalho Serafim. Overcoming treatment-resistant major depressive disorder outside of the doctor's office. Psychiatry Research 2019; 279: 397-398. |
| Wrong setting | Muke SS, Tugnawat D, Joshi U, et al. Digital Training for Non-Specialist Health Workers to Deliver a Brief Psychological Treatment for Depression in Primary Care in India: Findings from a Randomized Pilot Study. Int J Environ Res Public Health 2020; 17: . |
| Wrong patient population | Naik AD, Hundt NE, Vaughan EM, Petersen NJ, Zeno D, Kunik ME, Cully JA. Effect of Telephone-Delivered Collaborative Goal Setting and Behavioral Activation vs Enhanced Usual Care for Depression Among Adults With Uncontrolled Diabetes: A Randomized Clinical Trial. JAMA Netw Open 2019; 2: e198634. |
| Wrong study design | Nakao S, Nakagawa A, Oguchi Y, et al. Web-Based Cognitive Behavioral Therapy Blended With Face-to-Face Sessions for Major Depression: Randomized Controlled Trial. J Med Internet Res 2018; 20: e10743. |
| Not Randomized | Nelson CBeau, Abraham Kristen M, Walters Heather, Pfeiffer Paul N, Valenstein Marcia. Integration of peer support and computer-based CBT for veterans with depression. Computers in Human Behavior 2014; 31: 57-64. |
| Wrong patient population | Neubauer K, von Auer M, Murray E, Petermann F, Helbig-Lang S, Gerlach AL. Internet-delivered attention modification training as a treatment for social phobia: a randomized controlled trial. Behav Res Ther 2013; 51: 87-97. |
| Wrong patient population | Newby J, Robins L, Wilhelm K, et al. Web-Based Cognitive Behavior Therapy for Depression in People With Diabetes Mellitus: A Randomized Controlled Trial. J Med Internet Res 2017; 19: e157. |
| Wrong patient population | Newby JM, Mackenzie A, Williams AD, McIntyre K, Watts S, Wong N, Andrews G. Internet cognitive behavioural therapy for mixed anxiety and depression: a randomized controlled trial and evidence of effectiveness in primary care. Psychol Med 2013; 43: 2635-48. |
| Wrong patient population | Newby JM, Mewton L, Andrews G. Transdiagnostic versus disorder-specific internet-delivered cognitive behaviour therapy for anxiety and depression in primary care. Journal of Anxiety Disorders 2017; 46: 25-34. |
| Wrong patient population | Newby JM, Williams AD, Andrews G. Reductions in negative repetitive thinking and metacognitive beliefs during transdiagnostic internet cognitive behavioural therapy (iCBT) for mixed anxiety and depression. Behav Res Ther 2014; 59: 52-60. |
| Wrong patient population | Ngai FW, Wong PW, Leung KY, Chau PH, Chung KF. The Effect of Telephone-Based Cognitive-Behavioral Therapy on Postnatal Depression: A Randomized Controlled Trial. Psychother Psychosom 2015; 84: 294-303. |
| Wrong study design | Nicholas J, Knapp AA, Vergara JL, et al. An Exploratory Brief Head-To-Head Non-Inferiority Comparison of an Internet-Based and a Telephone-Delivered CBT Intervention for Adults with Depression. J Affect Disord 2021; 281: 673-677. |
| Wrong patient population | Nieminen K, Berg I, Frankenstein K, et al. Internet-provided cognitive behaviour therapy of posttraumatic stress symptoms following childbirth-a randomized controlled trial. Cogn Behav Ther 2016; 45: 287-306. |
| Wrong patient population | Niles AN, Axelsson E, Andersson E, et al. Internet-based cognitive behavior therapy for depression, social anxiety disorder, and panic disorder: Effectiveness and predictors of response in a teaching clinic. Behav Res Ther 2021; 136: 103767. |
| Wrong patient population | Nordgren LB, Hedman E, Etienne J, et al. Effectiveness and cost-effectiveness of individually tailored Internet-delivered cognitive behavior therapy for anxiety disorders in a primary care population: a randomized controlled trial. Behav Res Ther 2014; 59: 44572. |
| Wrong patient population | Nygren T, Brohede D, Koshnaw K, Osman SS, Johansson R, Andersson G. Internet-based treatment of depressive symptoms in a Kurdish population: A randomized controlled trial. J Clin Psychol 2019; 75: 985-998. |
| Wrong patient population | O'Mahen HA, Richards DA, Woodford J, Wilkinson E, McGinley J, Taylor RS, Warren FC. Netmums: a phase II randomized controlled trial of a guided Internet behavioural activation treatment for postpartum depression. Psychol Med 2014; 44: 1675-89. |
| Duplicate Study | O'Mahen HA, Wilkinson E, Bagnall K, Richards DA, Swales A. Shape of change in internet based behavioral activation treatment for depression. Behav Res Ther 2017; 95: 107-116. |
| Duplicate Study | O'Mahen HA, Woodford J, McGinley J, Warren FC, Richards DA, Lynch TR, Taylor RS. Internet-based behavioral activation--treatment for postnatal depression (Netmums): a randomized controlled trial. J Affect Disord 2013; 150: 814-22. |
| Wrong patient population | O'Moore K A, Newby JM, Andrews G, Hunter DJ, Bennell K, Smith J, Williams AD. Internet Cognitive‚ÄìBehavioral Therapy for Depression in Older Adults With Knee Osteoarthritis: A Randomized Controlled Trial. Arthritis Care and Research 2018; 70: 61-70. |
| Wrong patient population | O'Reilly H, Hagerty A, O'Donnell S, et al. Alcohol Use Disorder and Comorbid Depression: A Randomized Controlled Trial Investigating the Effectiveness of Supportive Text Messages in Aiding Recovery. Alcohol Alcohol 2019; 54: 551-558. |
| Wrong setting | Ofoegbu TO, Asogwa U, Otu MS, Ibenegbu C, Muhammed A, Eze B. Efficacy of guided internet-assisted intervention on depression reduction among educational technology students of Nigerian universities. Medicine (Baltimore) 2020; 99: e18774. |
| Wrong patient population | Oromendia P, Orrego J, Bonillo A, Molinuevo B. Internet-based self-help treatment for panic disorder: a randomized controlled trial comparing mandatory versus optional complementary psychological support. Cogn Behav Ther 2016; 45: 270-86. |
| Wrong patient population | Oser M, Wallace ML, Solano F, Szigethy EM. Guided Digital Cognitive Behavioral Program for Anxiety in Primary Care: Propensity-Matched Controlled Trial. JMIR Ment Health 2019; 6: e11981. |
| Wrong patient population | Otero P, Hita I, Torres √Å J, V√°zquez FL. Brief Psychological Intervention Through Mobile App and Conference Calls for the Prevention of Depression in Non-Professional Caregivers: A Pilot Study. Int J Environ Res Public Health 2020; 17: . |
| Wrong study design | Pabst A, L√∂bner M, Stein J, Luppa M, Kersting A, K√∂nig HH, Riedel-Heller SG. Internet-Based Cognitive Behavior Therapy Only for the Young? A Secondary Analysis of a Randomized Controlled Trial of Depression Treatment. Front Psychiatry 2020; 11: 735. |
| Wrong patient population | Palacios JE, Richards D, Palmer R, Coudray C, Hofmann SG, Palmieri PA, Frazier P. Supported Internet-Delivered Cognitive Behavioral Therapy Programs for Depression, Anxiety, and Stress in University Students: Open, Non-Randomised Trial of Acceptability, Effectiveness, and Satisfaction. JMIR Ment Health 2018; 5: e11467. |
| Wrong intervention | Pan KY, Kok AAL, Eikelenboom M, et al. The mental health impact of the COVID-19 pandemic on people with and without depressive, anxiety, or obsessive-compulsive disorders: a longitudinal study of three Dutch case-control cohorts. Lancet Psychiatry 2021; 8: 121-129. |
| Wrong patient population | Patel SR, Wheaton MG, Andersson E, et al. Acceptability, Feasibility, and Effectiveness of Internet-Based Cognitive-Behavioral Therapy for Obsessive-Compulsive Disorder in New York. Behav Ther 2018; 49: 631-641. |
| Wrong patient population | Paxling B, Almlov J, Dahlin M, Carlbring P, Breitholtz E, Eriksson T, Andersson G. Guided internet-delivered cognitive behavior therapy for generalized anxiety disorder: a randomized controlled trial. Cogn Behav Ther 2011; 40: 159-73. |
| Wrong outcomes | Perestelo-Perez L, Rivero-Santana A, Sanchez-Afonso JA, Perez-Ramos J, Castellano-Fuentes CL, Sepucha K, Serrano-Aguilar P. Effectiveness of a decision aid for patients with depression: A randomized controlled trial. Health Expect 2017; 20: 1096-1105. |
| Wrong study design | Pfeiffer Paul N, Valenstein Marcia, Ganoczy Dara, Henry Jennifer, Dobscha Steven K, Piette John D. Pilot study of enhanced social support with automated telephone monitoring after psychiatric hospitalization for depression. Social Psychiatry and Psychiatric Epidemiology: The International Journal for Research in Social and Genetic Epidemiology and Mental Health Services 2017; 52: 183-191. |
| Wrong patient population | Phillips R, Schneider J, Molosankwe I, et al. Randomized controlled trial of computerized cognitive behavioural therapy for depressive symptoms: effectiveness and costs of a workplace intervention. Psychol Med 2014; 44: 741-52. |
| High Risk of Bias | Pihlaja S, Lahti J, Lipsanen JO, Ritola V, Gummerus EM, Stenberg JH, Joffe G. Scheduled Telephone Support for Internet Cognitive Behavioral Therapy for Depression in Patients at Risk for Dropout: Pragmatic Randomized Controlled Trial. J Med Internet Res 2020; 22: e15732. |
| Wrong patient population | Pittaway S, Cupitt C, Palmer D, et al. Comparative, clinical feasibility study of three tools for delivery of cognitive behavioural therapy for mild to moderate depression and anxiety provided on a self-help basis. Mental Health in Family Medicine 2010; 6: 145-154. |
| Wrong patient population | Posmontier B, Neugebauer R, Stuart S, Chittams J, Shaughnessy R. Telephone-Administered Interpersonal Psychotherapy by Nurse-Midwives for Postpartum Depression. J Midwifery Womens Health 2016; 61: 456-66. |
| Wrong patient population | Possemato K, Johnson EM, Emery JB, et al. A pilot study comparing peer supported web-based CBT to self-managed web CBT for primary care veterans with PTSD and hazardous alcohol use. Psychiatr Rehabil J 2019; 42: 305-313. |
| Wrong patient population | Possemato K, Kuhn E, Johnson E, et al. Using PTSD Coach in primary care with and without clinician support: a pilot randomized controlled trial. Gen Hosp Psychiatry 2016; 38: 94-8. |
| Wrong patient population | Pots WT, Fledderus M, Meulenbeek PA, ten Klooster PM, Schreurs KM, Bohlmeijer ET. Acceptance and commitment therapy as a web-based intervention for depressive symptoms: randomised controlled trial. Br J Psychiatry 2016; 208: 69-77. |
| Wrong patient population | Powell J, Hamborg T, Stallard N, et al. Effectiveness of a web-based cognitive-behavioral tool to improve mental well-being in the general population: randomized controlled trial. J Med Internet Res 2012; 15: e2. |
| Wrong patient population | Proudfoot J, Clarke J, Birch MR, et al. Impact of a mobile phone and web program on symptom and functional outcomes for people with mild-to-moderate depression, anxiety and stress: a randomised controlled trial. BMC Psychiatry 2013; 13: 312. |
| Wrong patient population | Purkayastha S, Addepally SA, Bucher S. Engagement and Usability of a Cognitive Behavioral Therapy Mobile App Compared With Web-Based Cognitive Behavioral Therapy Among College Students: Randomized Heuristic Trial. JMIR Hum Factors 2020; 7: e14146. |
| Wrong outcomes | Pyne JM, Fortney JC, Mouden S, Lu L, Hudson TJ, Mittal D. Cost-effectiveness of on-site versus off-site collaborative care for depression in rural FQHCs. Psychiatr Serv 2015; 66: 491-9. |
| Wrong patient population | Pyne JM, Fortney JC, Tripathi SP, Maciejewski ML, Edlund MJ, Williams DK. Cost-effectiveness analysis of a rural telemedicine collaborative care intervention for depression. Arch Gen Psychiatry 2010; 67: 812-21. |
| Wrong patient population | Querstret D, Cropley M, Fife-Schaw C. The Effects of an Online Mindfulness Intervention on Perceived Stress, Depression and Anxiety in a Non-clinical Sample: A Randomised Waitlist Control Trial. Mindfulness (N Y) 2018; 9: 1825-1836. |
| Wrong type of publication | Reins JA, Ebert DD, Lehr D, Riper H, Cuijpers P, Berking M. Internet-based treatment of major depression for patients on a waiting list for inpatient psychotherapy: Protocol for a multi-centre randomised controlled trial. BMC Psychiatry 2013; 13: . |
| Non English | Ren Zhihong, Li Xianyun, Zhao Lingbo, et al. Effectiveness and mechanism of internet-based self-help intervention for depression: The Chinese version of MoodGYM. Acta Psychologica Sinica 2016; 48: 818-832. |
| Wrong study design | Richards D, Enrique A, Eilert N, et al. A pragmatic randomized waitlist-controlled effectiveness and cost-effectiveness trial of digital interventions for depression and anxiety. NPJ Digit Med 2020; 3: 85. |
| Wrong patient population | Richards D, Timulak L, O'Brien E, Hayes C, Vigano N, Sharry J, Doherty G. A randomized controlled trial of an internet-delivered treatment: Its potential as a low-intensity community intervention for adults with symptoms of depression. Behav Res Ther 2015; 75: 20-31. |
| Wrong study design | Richards DA, Borglin G. Implementation of psychological therapies for anxiety and depression in routine practice: Two year prospective cohort study. Journal of Affective Disorders 2011; 133: 51-60. |
| Wrong outcomes | Richards Derek, Timulak Ladislav, Hevey David. A comparison of two online cognitive-behavioural interventions for symptoms of depression in a student population: The role of therapist responsiveness. Counselling & Psychotherapy Research 2013; 13: 184-193. |
| Wrong patient population | Robinson E, Titov N, Andrews G, McIntyre K, Schwencke G, Solley K. Internet treatment for generalized anxiety disorder: a randomized controlled trial comparing clinician vs. technician assistance. PLoS One 2010; 5: e10942. |
| Study Design Only - No Results | Rodie DJ, Fitzgibbon K, Perivolaris A, et al. The primary care assessment and research of a telephone intervention for neuropsychiatric conditions with education and resources study: Design, rationale, and sample of the PARTNERs randomized controlled trial. Contemp Clin Trials 2021; 103: 106284. |
| Wrong patient population | Roepke AM, Jaffee SR, Riffle OM, McGonigal J, Broome R, Maxwell B. Randomized Controlled Trial of SuperBetter, a Smartphone-Based/Internet-Based Self-Help Tool to Reduce Depressive Symptoms. Games Health J 2015; 4: 235-46. |
| Wrong intervention | Rojas G, Guajardo V, Martinez P, Castro A, Fritsch R, Moessner M, Bauer S. A Remote Collaborative Care Program for Patients with Depression Living in Rural Areas: Open-Label Trial. J Med Internet Res 2018; 20: e158. |
| Wrong patient population | Rollman BL, Belnap BH, Mazumdar S, et al. Telephone-Delivered Stepped Collaborative Care for Treating Anxiety in Primary Care: A Randomized Controlled Trial. J Gen Intern Med 2017; 32: 245-255. |
| Wrong patient population | Rollman BL, Herbeck Belnap B, Abebe KZ, Spring MB, Rotondi AJ, Rothenberger SD, Karp JF. Effectiveness of Online Collaborative Care for Treating Mood and Anxiety Disorders in Primary Care: A Randomized Clinical Trial. JAMA Psychiatry 2018; 75: 56-64. |
| Wrong patient population | Rosen CS, Azevedo KJ, Tiet QQ, et al. An RCT of effects of telephone care management on treatment adherence and clinical outcomes among veterans with PTSD. Psychiatric Services 2017; 68: 151-158. |
| Wrong patient population | Rosen CS, Tiet QQ, Harris AH, et al. Telephone monitoring and support after discharge from residential PTSD treatment: a randomized controlled trial. Psychiatr Serv 2013; 64: 13-20. |
| Wrong timeline | Rosso Isabelle M, Killgore William DS, Olson Elizabeth A, et al. Internet‚Äêbased cognitive behavior therapy for major depressive disorder: A randomized controlled trial. Depression and Anxiety 2017; 34: 236-245. |
| Wrong patient population | Roy-Byrne P, Craske MG, Sullivan G, et al. Delivery of evidence-based treatment for multiple anxiety disorders in primary care: a randomized controlled trial. Jama 2010; 303: 1921-8. |
| Wrong outcomes | Ruggiero KJ, Price M, Adams Z, et al. Web Intervention for Adolescents Affected by Disaster: Population-Based Randomized Controlled Trial. J Am Acad Child Adolesc Psychiatry 2015; 54: 709-17. |
| Wrong patient population | Russell A, Gaunt D, Cooper K, et al. Guided self-help for depression in autistic adults: the ADEPT feasibility RCT. Health Technol Assess 2019; 23: 34335. |
| Wrong type of publication | Ruwaard Jeroen, Lange Alfred, Schrieken Bart, Emmelkamp Paul. Efficacy and effectiveness of online cognitive behavioral treatment: A decade of interapy research. Annual Review of CyberTherapy and Telemedicine 2011; 9: 44847. |
| Wrong study design | Ruzek JI, Wilk J, Simon E, et al. Randomized Controlled Trial of a Web-Based Intervention to Disseminate Clinical Practice Guidelines for Posttraumatic Stress Disorder: The PTSD Clinicians Exchange. J Trauma Stress 2020; : . |
| Wrong patient population | Salamanca-Sanabria A, Richards D, Timulak L, Connell S, Mojica Perilla M, Parra-Villa Y, Castro-Camacho L. A Culturally Adapted Cognitive Behavioral Internet-Delivered Intervention for Depressive Symptoms: Randomized Controlled Trial. JMIR Ment Health 2020; 7: e13392. |
| Wrong intervention | Salemink E, Kindt M, Rienties H, van den Hout M. Internet-based cognitive bias modification of interpretations in patients with anxiety disorders: a randomised controlled trial. J Behav Ther Exp Psychiatry 2014; 45: 186-95. |
| Wrong intervention | Salisbury C, O'Cathain A, Edwards L, et al. Effectiveness of an integrated telehealth service for patients with depression: a pragmatic randomised controlled trial of a complex intervention. Lancet Psychiatry 2016; 3: 515-25. |
| Wrong patient population | Schlicker S, Baumeister H, Buntrock C, et al. A Web- and Mobile-Based Intervention for Comorbid, Recurrent Depression in Patients With Chronic Back Pain on Sick Leave (Get.Back): Pilot Randomized Controlled Trial on Feasibility, User Satisfaction, and Effectiveness. JMIR Ment Health 2020; 7: e16398. |
| Wrong indication | Schlicker S, Ebert DD, Middendorf T, Titzler I, Berking M. Evaluation of a text-message-based maintenance intervention for Major Depressive Disorder after inpatient cognitive behavioral therapy. J Affect Disord 2018; 227: 305-312. |
| Wrong outcomes | Schlicker S, Weisel KK, Buntrock C, et al. Do Nonsuicidal Severely Depressed Individuals with Diabetes Profit from Internet-Based Guided Self-Help? Secondary Analyses of a Pragmatic Randomized Trial. J Diabetes Res 2019; 2019: 2634094. |
| Wrong patient population | Schlosser DA, Campellone TR, Truong B, Anguera JA, Vergani S, Vinogradov S, Arean P. The feasibility, acceptability, and outcomes of PRIME-D: A novel mobile intervention treatment for depression. Depress Anxiety 2017; 34: 546-554. |
| Wrong intervention | Schm√§deke Stefan, Bischoff Claus. Wirkungen smartphonegest√ºtzter psychosomatischer Rehabilitationsnachsorge (eATROS) bei depressiven Patienten = Effects of smartphone-supported rehabilitation aftercare (eATROS) for depressive patients. Verhaltenstherapie 2015; 25: 277-286. |
| Wrong patient population | Schneider J, Sarrami Foroushani P, Grime P, Thornicroft G. Acceptability of online self-help to people with depression: users' views of MoodGYM versus informational websites. J Med Internet Res 2014; 16: e90. |
| Wrong patient population | Schotanus-Dijkstra Marijke, Pieterse Marcel E, Drossaert Constance HC, Walburg Jan A, Bohlmeijer Ernst T. Possible mechanisms in a multicomponent email guided positive psychology intervention to improve mental well-being, anxiety and depression: A multiple mediation model. The Journal of Positive Psychology 2019; 14: 141-155. |
| Wrong study design | Schuster R, Laireiter AR, Berger T, Moritz S, Meyer B, Hohagen F, Klein JP. Immediate and long-term effectiveness of adding an Internet intervention for depression to routine outpatient psychotherapy: Subgroup analysis of the EVIDENT trial. J Affect Disord 2020; 274: 643-651. |
| Wrong patient population | Sethi Suvena. Treating youth depression and anxiety: A randomised controlled trial examining the efficacy of computerised versus face‚Äêto‚Äêface cognitive behaviour therapy. Australian Psychologist 2013; 48: 249-257. |
| Wrong patient population | Sheeber LB, Feil EG, Seeley JR, et al. Mom-net: Evaluation of an internet-facilitated cognitive behavioral intervention for low-income depressed mothers. J Consult Clin Psychol 2017; 85: 355-366. |
| Wrong patient population | Sheeber LB, Seeley JR, Feil EG, Davis B, Sorensen E, Kosty DB, Lewinsohn PM. Development and pilot evaluation of an Internet-facilitated cognitive-behavioral intervention for maternal depression. J Consult Clin Psychol 2012; 80: 739-749. |
| Wrong patient population | Silfvernagel K, Carlbring P, Kabo J, Edstrom S, Eriksson J, Manson L, Andersson G. Individually tailored internet-based treatment for young adults and adults with panic attacks: randomized controlled trial. J Med Internet Res 2012; 14: e65. |
| Wrong indication | Silfvernagel K, Westlinder A, Andersson S, et al. Individually tailored internet-based cognitive behaviour therapy for older adults with anxiety and depression: a randomised controlled trial. Cogn Behav Ther 2018; 47: 286-300. |
| Wrong intervention | Simons AD, Padesky CA, Montemarano J, et al. Training and dissemination of cognitive behavior therapy for depression in adults: a preliminary examination of therapist competence and client outcomes. J Consult Clin Psychol 2010; 78: 751-6. |
| Wrong patient population | Spence J, Titov N, Johnston L, Jones MP, Dear BF, Solley K. Internet-based trauma-focused cognitive behavioural therapy for PTSD with and without exposure components: a randomised controlled trial. J Affect Disord 2014; 162: 73-80. |
| Wrong patient population | Srivastava Paakhi, Mehta Manju, Sagar Rajesh, Ambekar Atul. Smartteen- a computer assisted cognitive behavior therapy for Indian adolescents with depression- a pilot study. Asian Journal of Psychiatry 2020; 50: . |
| Wrong patient population | Staples LG, Fogliati VJ, Dear BF, Nielssen O, Titov N. Internet-delivered treatment for older adults with anxiety and depression: implementation of the Wellbeing Plus Course in routine clinical care and comparison with research trial outcomes. BJPsych Open 2016; 2: 307-313. |
| Wrong patient population | Stecker T, McHugo G, Xie H, Whyman K, Jones M. RCT of a brief phone-based CBT intervention to improve PTSD treatment utilization by returning service members. Psychiatr Serv 2014; 65: 1232-7. |
| Wrong patient population | Steinmetz SE, Benight CC, Bishop SL, James LE. My Disaster Recovery: a pilot randomized controlled trial of an Internet intervention. Anxiety Stress Coping 2012; 25: 593-600. |
| Wrong patient population | Stiles-Shields C, Montague E, Kwasny MJ, Mohr DC. Behavioral and cognitive intervention strategies delivered via coached apps for depression: Pilot trial. Psychol Serv 2019; 16: 233-238. |
| Wrong patient population | Strachan M, Gros DF, Ruggiero KJ, Lejuez CW, Acierno R. An integrated approach to delivering exposure-based treatment for symptoms of PTSD and depression in OIF/OEF veterans: preliminary findings. Behav Ther 2012; 43: 560-9. |
| Wrong patient population | Strauss C, Dunkeld C, Cavanagh K. Is clinician-supported use of a mindfulness smartphone app a feasible treatment for depression? A mixed-methods feasibility study. Internet Interventions 2021; 25: . |
| Wrong intervention | Strom M, Uckelstam CJ, Andersson G, Hassmen P, Umefjord G, Carlbring P. Internet-delivered therapist-guided physical activity for mild to moderate depression: a randomized controlled trial. PeerJ 2013; 1: e178. |
| Wrong patient population | Stubbings Daniel R, Rees Clare S, Roberts Lynne D, Kane Robert T. Comparing in-person to videoconference-based cognitive behavioral therapy for mood and anxiety disorders: Randomized controlled trial. Journal of Medical Internet Research 2013; 15: 169-184. |
| Wrong intervention | Sugg HVR, Richards DA, Frost J. Morita Therapy for depression (Morita Trial): a pilot randomised controlled trial. BMJ Open 2018; 8: e021605. |
| Wrong intervention | Sweet AM, Pearlstein SL, Paulus MP, Stein MB, Taylor CT. Computer-delivered behavioural activation and approach-avoidance training in major depression: Proof of concept and initial outcomes. Br J Clin Psychol 2021; 60: 357-374. |
| Wrong outcomes | T√∂nnies J, Hartmann M, Wensing M, et al. Mental health specialist video consultations versus treatment-as-usual for patients with depression or anxiety disorders in primary care: Randomized controlled feasibility trial. JMIR Mental Health 2021; 8: . |
| Wrong study design | T√∏nning ML, Faurholt-Jepsen M, Frost M, et al. The effect of smartphone-based monitoring and treatment on the rate and duration of psychiatric readmission in patients with unipolar depressive disorder: The RADMIS randomized controlled trial. J Affect Disord 2021; 282: 354-363. |
| Wrong study design | Thase ME, McCrone P, Barrett MS, et al. Improving Cost-effectiveness and Access to Cognitive Behavior Therapy for Depression: Providing Remote-Ready, Computer-Assisted Psychotherapy in Times of Crisis and Beyond. Psychother Psychosom 2020; 89: 307-313. |
| Wrong setting | Thitipitchayanant K, Somrongthong R, Kumar R, Kanchanakharn N. Effectiveness of self-empowerment-affirmation-relaxation (Self-EAR) program for postpartum blues mothers: A randomize controlled trial. Pak J Med Sci 2018; 34: 1488-1493. |
| Duplicate Study | Titov N, Andrews G, Davies M, McIntyre K, Robinson E, Solley K. Internet treatment for depression: a randomized controlled trial comparing clinician vs. technician assistance. PLoS One 2010; 5: e10939. |
| Wrong patient population | Titov N, Dear BF, Ali S, et al. Clinical and cost-effectiveness of therapist-guided internet-delivered cognitive behavior therapy for older adults with symptoms of depression: a randomized controlled trial. Behav Ther 2015; 46: 193-205. |
| Wrong patient population | Titov N, Dear BF, Johnston L, et al. Improving adherence and clinical outcomes in self-guided internet treatment for anxiety and depression: a 12-month follow-up of a randomised controlled trial. PLoS One 2014; 9: e89591. |
| Duplicate Study | Titov N, Dear BF, Johnston L, et al. Improving adherence and clinical outcomes in self-guided internet treatment for anxiety and depression: randomised controlled trial. PLoS One 2013; 8: e62873. |
| Wrong patient population | Titov N, Dear BF, Schwencke G, Andrews G, Johnston L, Craske MG, McEvoy P. Transdiagnostic internet treatment for anxiety and depression: a randomised controlled trial. Behav Res Ther 2011; 49: 441-52. |
| Wrong patient population | Titov N, Dear BF, Staples LG, et al. Disorder-specific versus transdiagnostic and clinician-guided versus self-guided treatment for major depressive disorder and comorbid anxiety disorders: A randomized controlled trial. J Anxiety Disord 2015; 35: 88-102. |
| Wrong patient population | Titov N, Fogliati VJ, Staples LG, et al. Treating anxiety and depression in older adults: randomised controlled trial comparing guided v. self-guided internet-delivered cognitive-behavioural therapy. BJPsych Open 2016; 2: 50-58. |
| Wrong patient population | Tomasino KN, Lattie EG, Ho J, Palac HL, Kaiser SM, Mohr DC. Harnessing Peer Support in an Online Intervention for Older Adults with Depression. Am J Geriatr Psychiatry 2017; 25: 1109-1119. |
| Wrong patient population | Travis J, Roeder K, Walters H, et al. Telephone-based mutual peer support for depression: a pilot study. Chronic Illn 2010; 6: 183-91. |
| Wrong study design | Trombello JM, South C, S√°nchez A, Kahalnik F, Kennard BD, Trivedi MH. Two Trajectories of Depressive Symptom Reduction Throughout Behavioral Activation Teletherapy Among Underserved, Ethnically Diverse, Primary Care Patients: A VitalSign6 Report. Behavior Therapy 2020; : . |
| Wrong intervention | Truzoli Roberto, Rovetta Cecilia, Nola Eliana, Matteucci Luca, Vigan√≤ Caterina. Effectiveness of text messaging for the management of psychological and somatic distress in depressed and anxious outpatients. The Open Psychology Journal 2019; 12: . |
| Wrong study design | Tulbure BT, Andersson G, Salagean N, Pearce M, Koenig HG. Religious versus Conventional Internet-based Cognitive Behavioral Therapy for Depression. J Relig Health 2018; 57: 1634-1648. |
| Wrong patient population | Tulbure BT, Rusu A, Sava FA, Salagean N, Farchione TJ. A Web-Based Transdiagnostic Intervention for Affective and Mood Disorders: Randomized Controlled Trial. JMIR Ment Health 2018; 5: e36. |
| Not original research | Tyrer P, Cooper S, Salkovskis P, et al.. Erratum: Clinical and cost-effectiveness of cognitive behaviour therapy for health anxiety in medical patients: a multicentre randomised controlled trial (The Lancet (2014) 383(9913) (219‚Äì225) (S0140673613619054) (10.1016/S0140-6736(13)61905-4)). The Lancet 2014; 383: 218. |
| Wrong comparator | Uebelacker L, Dufour SC, Dinerman JG, et al. Examining the Feasibility and Acceptability of an Online Yoga Class for Mood Disorders: A MoodNetwork Study. Journal of Psychiatric Practice 2018; 24: 60-67. |
| Wrong intervention | Uebelacker LA, Marootian BA, Tigue P, Haggarty R, Primack JM, Miller IW. Telephone depression care management for Latino Medicaid health plan members: a pilot randomized controlled trial. J Nerv Ment Dis 2011; 199: 678-83. |
| Study Design Only - No Results | V√°zquez FL, Torres A, D√≠az O, P√°ramo M, Otero P, Blanco V, L√≥pez L. Cognitive behavioral intervention via a smartphone app for non-professional caregivers with depressive symptoms: Study protocol for a randomized controlled trial. Trials 2018; 19: . |
| Wrong intervention | Van Lieshout RJ, Layton H, et al. Effect of Online 1-Day Cognitive Behavioral Therapy-Based Workshops Plus Usual Care vs Usual Care Alone for Postpartum Depression: A Randomized Clinical Trial. JAMA Psychiatry 2021; : . |
| Wrong study design | Vara MD, Mira A, Miragall M, et al. A Low-Intensity Internet-Based Intervention Focused on the Promotion of Positive Affect for the Treatment of Depression in Spanish Primary Care: Secondary Analysis of a Randomized Controlled Trial. Int J Environ Res Public Health 2020; 17: . |
| Not Randomized | Venkatesan A, Rahimi L, Kaur M, Mosunic C. Digital cognitive behavior therapy intervention for depression and anxiety: Retrospective study. JMIR Mental Health 2020; 7: . |
| Wrong patient population | Vervaeke J, Hoorelbeke K, Baeken C, Koster Ernst HW. Online cognitive control training for remitted depressed individuals: A replication and extension study. Cognitive Therapy and Research 2021; 45: 944-958. |
| Wrong patient population | Voderholzer U, Beintner I, Backes B, Esguerra E, Hessler-Kaufmann JB. Implementing Videoconference CBT for Depression in Routine Outpatient Care: Outcome, Working Alliance, and Influence of Patients' Technology Commitment. Verhaltenstherapie 2021; : . |
| Wrong patient population | Wagner B, Horn AB, Maercker A. Internet-based versus face-to-face cognitive-behavioral intervention for depression: a randomized controlled non-inferiority trial. J Affect Disord 2014; 152-154: 113-21. |
| Wrong patient population | Wahbeh H. Internet Mindfulness Meditation Intervention (IMMI) Improves Depression Symptoms in Older Adults. Medicines (Basel) 2018; 5: . |
| Wrong intervention | Wahle F, Kowatsch T, Fleisch E, Rufer M, Weidt S. Mobile Sensing and Support for People With Depression: A Pilot Trial in the Wild. JMIR Mhealth Uhealth 2016; 4: e111. |
| Wrong patient population | Wang JP, Maercker A. Web-based interventions for traumatized people in mainland China. Eur J Psychotraumatol 2014; 5: 26519. |
| High Risk of Bias | Watts S, Mackenzie A, Thomas C, Griskaitis A, Mewton L, Williams A, Andrews G. CBT for depression: a pilot RCT comparing mobile phone vs. computer. BMC Psychiatry 2013; 13: 49. |
| Wrong outcomes | Weisel KK, Zarski AC, Berger T, et al. Efficacy and cost-effectiveness of guided and unguided internet- and mobile-based indicated transdiagnostic prevention of depression and anxiety (ICare Prevent): A three-armed randomized controlled trial in four European countries. Internet Interv 2019; 16: 52-64. |
| Wrong intervention | Wierwille JL, Pukay-Martin ND, Chard KM, Klump MC. Effectiveness of PTSD telehealth treatment in a VA clinical sample. Psychol Serv 2016; 13: 373-379. |
| Wrong study design | Williams AD, Andrews G. The effectiveness of Internet cognitive behavioural therapy (iCBT) for depression in primary care: a quality assurance study. PLoS One 2013; 8: e57447. |
| Wrong intervention | Williams AD, Blackwell SE, Mackenzie A, Holmes EA, Andrews G. Combining imagination and reason in the treatment of depression: a randomized controlled trial of internet-based cognitive-bias modification and internet-CBT for depression. J Consult Clin Psychol 2013; 81: 793-9. |
| Wrong outcomes | Williams C, McClay CA, Martinez R, Morrison J, Haig C, Jones R, Farrand P. Online CBT life skills programme for low mood and anxiety: Study protocol for a pilot randomized controlled trial. Trials 2016; 17: . |
| Wrong patient population | Wilson M, Hewes C, Barbosa-Leiker C, Mason A, Wuestney KA, Shuen JA, Wilson MP. Engaging Adults With Chronic Disease in Online Depressive Symptom Self-Management. West J Nurs Res 2018; 40: 834-853. |
| Wrong patient population | Wisner KL, Sit DKY, McShea M, et al. Telephone-Based Depression Care Management for Postpartum Women: A Randomized Controlled Trial. J Clin Psychiatry 2017; 78: 1369-1375. |
| Wrong outcomes | Witlox M, Kraaij V, Garnefski N, et al. An Internet-based Acceptance and Commitment Therapy intervention for older adults with anxiety complaints: Study protocol for a cluster randomized controlled trial 11 Medical and Health Sciences 1117 Public Health and Health Services 11 Medical and Health Sciences 1103 Clinical Sciences 17 Psychology and Cognitive Sciences 1701 Psychology. Trials 2018; 19: . |
| Wrong patient population | Wootton BM, Dear BF, Johnston L, Terides MD, Titov N. Remote treatment of obsessive-compulsive disorder: A randomized controlled trial. Journal of Obsessive-Compulsive and Related Disorders 2013; 2: 375-384. |
| Duplicate Study | Wozney L, Olthuis J, Lingley-Pottie P, et al. Strongest Families‚Ñ¢ Managing Our Mood (MOM): a randomized controlled trial of a distance intervention for women with postpartum depression. Archives of Women's Mental Health 2017; 20: 525-537. |
| Wrong patient population | Yang R, Vigod SN, Hensel JM. Optional Web-Based Videoconferencing Added to Office-Based Care for Women Receiving Psychotherapy During the Postpartum Period: Pilot Randomized Controlled Trial. J Med Internet Res 2019; 21: e13172. |
| Wrong indication | Yeung A, Martinson MA, Baer L, et al. The Effectiveness of Telepsychiatry-Based Culturally Sensitive Collaborative Treatment for Depressed Chinese American Immigrants: A Randomized Controlled Trial. J Clin Psychiatry 2016; 77: e996-e1002. |
| Wrong intervention | Yeung A, Wang F, Feng F, et al. Outcomes of an online computerized cognitive behavioral treatment program for treating chinese patients with depression: A pilot study. Asian J Psychiatr 2018; 38: 102-107. |
| Wrong patient population | Yuen EK, Gros DF, Price M, Zeigler S, Tuerk PW, Foa EB, Acierno R. Randomized Controlled Trial of Home-Based Telehealth Versus In-Person Prolonged Exposure for Combat-Related PTSD in Veterans: Preliminary Results. J Clin Psychol 2015; 71: 500-12. |
| Wrong study design | Yusim Anna, Grigaitis Justinas. Efficacy of binaural beat meditation technology for treating anxiety symptoms: A pilot study. Journal of Nervous and Mental Disease 2020; 208: 155-160. |
| Wrong patient population | Zagorscak P, Heinrich M, Sommer D, Wagner B, Knaevelsrud C. Benefits of Individualized Feedback in Internet-Based Interventions for Depression: A Randomized Controlled Trial. Psychother Psychosom 2018; 87: 32-45. |
| Wrong intervention | Zanjani F, Bush H, Oslin D. Telephone-based psychiatric referral-care management intervention health outcomes. Telemed J E Health 2010; 16: 543-50. |
| Wrong intervention | Ziemba SJ, Bradley NS, Landry LA, Roth CH, Porter LS, Cuyler RN. Posttraumatic stress disorder treatment for Operation Enduring Freedom/Operation Iraqi Freedom combat veterans through a civilian community-based telemedicine network. Telemed J E Health 2014; 20: 446-50. |
| Wrong patient population | Zimmerman M, Terrill D, D'Avanzato C, Tirpak JW. Telehealth Treatment of Patients in an Intensive Acute Care Psychiatric Setting During the COVID-19 Pandemic: Comparative Safety and Effectiveness to In-Person Treatment. J Clin Psychiatry 2021; 82: . |
| Wrong study design | Zou JB, Dear BF, Titov N, et al. Brief internet-delivered cognitive behavioral therapy for anxiety in older adults: a feasibility trial. J Anxiety Disord 2012; 26: 650-5. |
| Duplicate Study | Zwerenz R, Baumgarten C, Becker J, Tibubos A, Siepmann M, Knickenberg RJ, Beutel ME. Improving the Course of Depressive Symptoms After Inpatient Psychotherapy Using Adjunct Web-Based Self-Help: Follow-Up Results of a Randomized Controlled Trial. J Med Internet Res 2019; 21: e13655. |
| Wrong study design | Zwerenz R, Becker J, Knickenberg RJ, Siepmann M, Hagen K, Beutel ME. Online Self-Help as an Add-On to Inpatient Psychotherapy: Efficacy of a New Blended Treatment Approach. Psychother Psychosom 2017; 86: 341-350. |
